# Photoperiod response-related gene *SiCOL1* contributes to flowering in sesame

**DOI:** 10.1186/s12870-018-1583-z

**Published:** 2018-12-10

**Authors:** Rong Zhou, Pan Liu, Donghua Li, Xiurong Zhang, Xin Wei

**Affiliations:** 1 Key Laboratory of Biology and Genetic Improvement of Oil Crops of the Ministry of Agriculture, Oil Crops Research Institute of the Chinese Academy of Agricultural Sciences, Wuhan, 430062 China; 2 College of Life Sciences, Shanghai Normal University, Shanghai, 200234 China

**Keywords:** Sesame, Photoperiod response, Flowering, Artificial selection, *CONSTANS*

## Abstract

**Background:**

Sesame is a major oilseed crop which is widely cultivated all around the world. Flowering, the timing of transition from vegetative to reproductive growth, is one of the most important events in the life cycle of sesame. Sesame is a typical short-day (SD) plant and its flowering is largely affected by photoperiod. However, the flowering mechanism in sesame at the molecular level is still not very clear. Previous studies showed that the *CONSTANS* (*CO*) gene is the crucial photoperiod response gene which plays a center role in duration of the plant vegetative growth.

**Results:**

In this study, the *CO*-*like* (*COL)* genes were identified and characterized in the sesame genome. Two homologs of the *CO* gene in the *SiCOLs*, *SiCOL1* and *SiCOL2*, were recognized and comprehensively analyzed. However, sequence analysis showed that *SiCOL2* lacked one of the B-box motifs. In addition, the flowering time of the transgenic *Arabidopsis* lines with overexpressed *SiCOL2* were longer than that of *SiCOL1*, indicating that *SiCOL1* was more likely to be the potential functional homologue of *CO* in sesame. Expression analysis revealed that *SiCOL1* had high expressed levels before flowering in leaves and exhibited diurnal rhythmic expression in both SD and long-day (LD) conditions. In total, 16 haplotypes of *SiCOL1* were discovered in the sesame collections from Asia. However, the mutated haplotypes did not express under both SD and LD conditions and was regarded as a nonfunctional allele. Notably, the sesame landraces from high-latitude regions harboring nonfunctional alleles of *SiCOL1* flowered much earlier than landraces from low-latitude regions under LD condition, and adapted to the northernmost regions of sesame cultivation. The result indicated that sesame landraces from high-latitude regions might have undergone artificial selection to adapt to the LD environment.

**Conclusions:**

Our results suggested that *SiCOL1* might contribute to regulation of flowering in sesame and natural variations in *SiCOL1* were probably related to the expansion of sesame cultivation to high-latitude regions. The results could be used in sesame breeding and in broadening adaptation of sesame varieties to new regions.

**Electronic supplementary material:**

The online version of this article (10.1186/s12870-018-1583-z) contains supplementary material, which is available to authorized users.

## Background

Flowering, the timing of transition from vegetative to reproductive development, is one of the critical developmental steps in plants. Previous research revealed that flowering is regulated by both genotype and environmental factors such as temperature, light spectrum, light intensity and day length (photoperiod). For example, winter wheat required several weeks at low temperature, also named vernalization to flower [1]. Flowering of rice is promoted in short photoperiod, and it is therefore regarded as a facultative SD plant [2], while flowering of *Arabidopsis thaliana* is mainly promoted by LD condition [3]. Recently, low red light to far-red light ratio was also reported to accelerate *Arabidopsis* flowering [4]. Flowering of *Phalaenopsis* is positively influenced by supplemental lighting during the inductive phase [5]. Among these environment factors that are related to plant flowering, photoperiod might be the critical signal that regulate the initiation of flowering in angiosperms since day length is a reliable indicator of the time of year for plants [6].

Flowering time is the key trait for geographical and seasonal adaptation in crops. For the worldwide cultivated crops, such as rice and soybean, the flowering time of varieties varied in a broad range and is related to yield. In a reasonable range, the late flowering, also means longer vegetative growth, contributes to the higher biomass and yield of the varieties [7, 8]. Usually, the varieties of SD plants from low latitude areas flower later than those from high latitude areas when the cultivars are planted under LD condition. To adapt the day-light conditions in different environments, the flowering time of crops would be selected during long-time breeding programs. Therefore, the flowering time and photoperiod sensitivity of crops are one of the primary improvement targets for crop breeding.

Sesame (*Sesamum indicum* L.), which belongs to the genus *Sesamum* in the family Pedaliaceae, is an important oilseed crop grown widely in various areas of the world. The harvest area of sesame has been doubled in the last four decades, and is still increasing gradually (http://www.fao.org/faostat/). Sesame was domesticated from wild relatives in the Indian subcontinent about 5000 years ago and it is therefore regarded as the most ancient oilseed crop [9, 10]. Nowadays, sesame is widely planted in more than 80 countries across the world, with a concentration in tropical and subtropical areas. Sesame flowering is mainly promoted by short photoperiod and is classified as a SD plant [11, 12]. The flowering time of sesame landraces ranges broadly from less than 30 d to more than 90 d [13]. However, few genetic researches of the sesame flowering have been reported and the genetic mechanism remains unknown.

The photoperiod gene, *CO*, encoding a B-box zinc-finger transcription factor, plays a central role in the photoperiod response and flowering regulation in *Arabidopsis* [3]. It acts between the circadian clock and florigen genes. The rhythmical expression of *CO* is regulated by *GI*, a circadian clock gene [14]. The peak expression of *CO* generally appears before dawn under SDs but at both afternoon and dawn under LDs [15]. Subsequently, *CO* activates expressions of the floral gene *FLOWERING LOCUS T* (*FT*), and promote flowering of *Arabidopsis* in LDs. It has been shown that CO protein binds to the *FT* promoter [16]. In LDs, the CO protein accumulates to higher levels due to the stability of the GI-FKF1 complex in the light which degrades *CO* repressor *CDF1* [17]. Although *CO* was identified in the LD plant *Arabidopsis*, its homologous genes in SD plants were also found to be the key flowering regulators. *Heading date 1* (*Hd1*), the homologous gene of *CO* in rice, contributes to photoperiod measurement and photoperiod-specific regulation of *FT* [18]. In contrast to *CO*, *Hd1* appears to be a bifunctional regulator, promoting *FT* expression in SDs but repressing *FT* in LDs [19, 20]. Moreover, the homologs of *CO* have been investigated in a number of other species, such as wheat, maize, barley, cotton, rapeseed, soybean, potato, grapevine, apple and *Pharbitis nil* through various functional genomics analyses, showing its conserved function involved in regulating plant flowering [21–30].

Previous studies showed *CO* had plenty of allelic variations, mediating photoperiod-dependent flowering time in *Arabidopsis* [31]. Among the 51 flowering time loci in *Arabidopsis*, *CO* possessed the most significantly associated single nucleotide polymorphisms (SNPs) of flowering time [32]. Similarly, a highly degree of polymorphisms of *Hd1* were the major determinant of the variation in flowering time diversity in rice [33]. Some variations of *CO* and *Hd1* had been identified as the crucial mutations that strongly influenced flowering time in plants [34, 35]. *COL* genes were also identified from many plants [22, 23, 30, 36–40]. B-box motifs and CCT domain have been proved to be the conserved domains in *COL* genes [23, 29, 41, 42]. However, the functional variations of *CO* and *COL* genes in sesame have not been identified and investigated.

Genome sequencing and large-scale genome re-sequencing of sesame has been completed recently [13, 43–45], providing high-quality reference genome sequence and massive useful variations for the functional genomics research of sesame. In the present study, the sesame *COL* gene family was genome-wide identified and characterized from the sesame genome. Two homologous genes of *CO* in sesame, *SiCOL1* and *SiCOL2*, were recognized. Functions of *SiCOL1* were confirmed by the transgenic approach, expression pattern analysis, and haplotype analysis. Evolution analysis of *SiCOL1* revealed that these genes had been selected to adapt to the photoperiod conditions in different areas. The results suggest that *SiCOL1* is an important agronomic photoperiod response gene that significantly affected flowering time, contributing to the adaption of sesame to the high-latitude regions. Our results also shed light on the potential value of *SiCOL1* in genetic improvement of sesame.

## Results

### Identification of *COL* genes in sesame

To identify the *COL* genes in sesame, the Hidden Markov Model (HMM) search was performed against the sesame protein database using the Zinc-finger B-box motif (PF00643) and CCT (*CONSTANS*, *CONSTANS*-*like*, *TIMING OF CAB EXPRESSION 1*) domain (PF06203). In total, 37 B-box Zinc-finger genes and 36 CCT domain-containing genes were identified in the sesame genome, respectively (Additional file 1: Table S1). The B-box Zinc-finger genes and the CCT domain-containing genes were then compared with each other and 13 genes of them were found to be the same. Therefore, the 13 genes which contained both Zinc-finger B-box motif and CCT domain were identified and named as sesame *COL* genes (Table 1). All of the *Arabidopsis* COL protein sequences were used as queries for the Basic Local Alignment Search Tool (BLAST) to identify sesame COL proteins. However, we had not identified any additional proteins containing both B-box motifs and CCT domain in the sesame genome. All B-box motif and CCT domain in the *SiCOLs* were validated by the CDD (http://www.ncbi.nlm.nih.gov/cdd/) and simple modular architecture research tool (SMART) analyses.

**Table 1:** The *COL* gene family in sesame

Gene	Linkage group	Position	Peptide length	Exon number	Group
SIN_1003782	LG16	4,171,550–4,176,377	415	4	III
SIN_1004896^b^	LG15	2,534,501–2,535,610	332	2	I
SIN_1011796	LG5	17,536,289–17,537,482	368	2	I
SIN_1012499	LG6	1,093,823–1,096,564	371	4	III
SIN_1013665	LG1	10,021,319–10,023,171	439	2	II
SIN_1017370	LG3	11,310,021–11,311,664	393	2	II
SIN_1018340	LG2	16,108,396–16,110,105	461	2	II
SIN_1019767	LG8	1,838,327–1,845,107	410	4	III
SIN_1019889^a^	LG8	2,936,072-2,937,312	354	2	I
SIN_1019954	LG5	13,943,158–13,945,055	412	4	III
SIN_1020629	LG6	18,213,252–18,214,375	339	2	I
SIN_1021657	LG1	930,153–932,165	335	2	I
SIN_1023877	LG2	9,655,751–9,656,982	378	2	I

^a^ *SiCOL1*

^b^ *SiCOL2*

The *SiCOL* genes were not evenly distributed on the linkage groups (LGs) of the sesame genome: one gene on LG3, LG15 and LG16, and two genes on LG1, LG2, LG5, LG6 and LG8. The SiCOL proteins ranged from 332 (*SIN_1004896*) to 461 (*SIN_1018340*) amino acids (aa) in length, with an average length of approximately 385 aa. Moreover, no tandem duplicate genes were identified for these *SiCOLs*, although tandem duplication events had been observed in several other sesame gene families [46–48].

### Phylogenetic analysis of the *SiCOL* genes

**Fig. 1:**
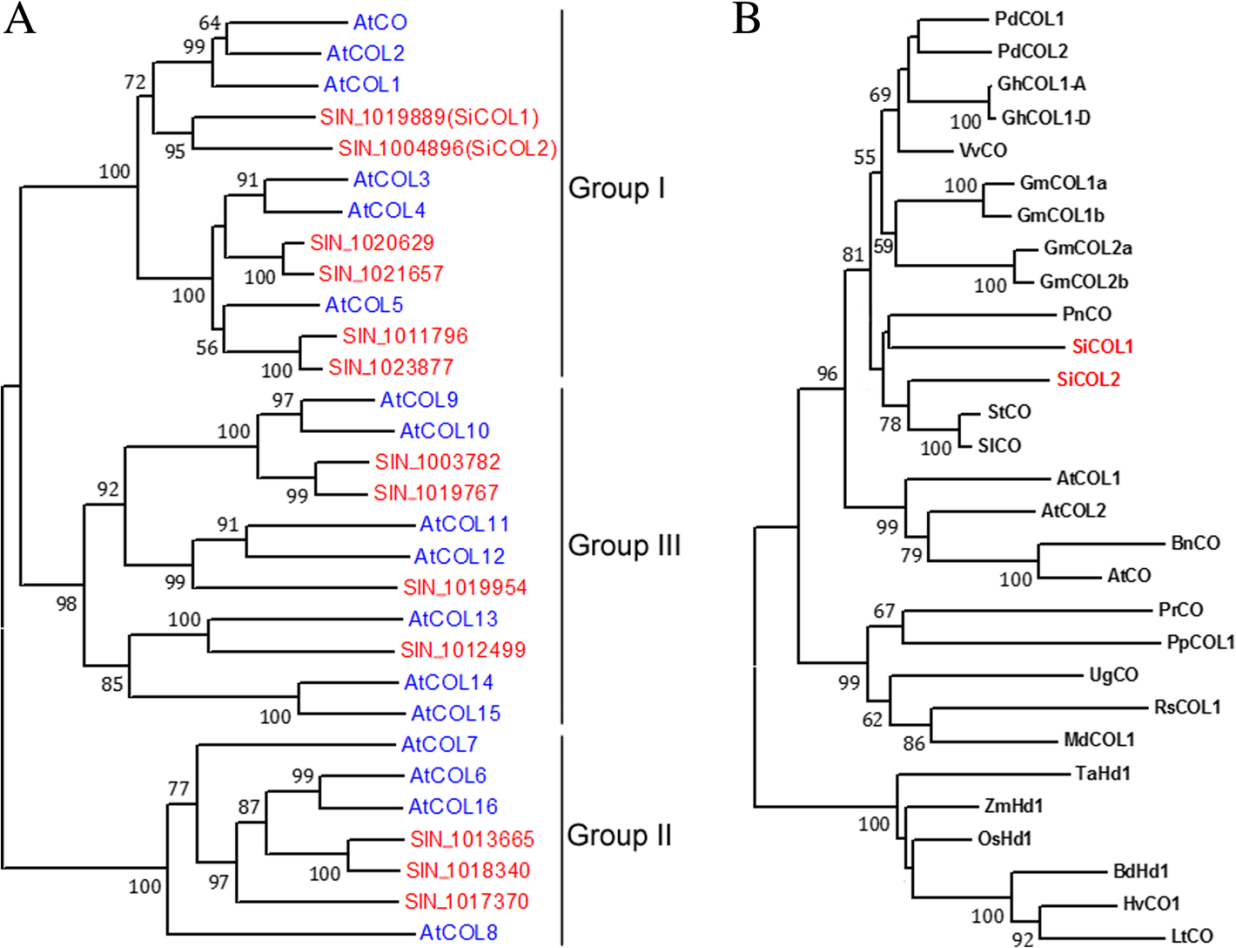
Phylogenetic analysis of the *SiCOL* genes. **a** A NJ phylogenetic tree of the COL proteins in sesame and *Arabidopsis*. The bootstrap values were inferred from 1000 replicates. **b** Phylogenetic relationship among COL proteins. The phylogram was generated from the multiple alignments of the deduced amino acid sequence from SiCOL1 and SiCOL2 and homologous proteins from other plant species. Bootstrap values from 1000 replicates were used to assess the robustness of the tree and the bootstrap values > 50% are showed

A phylogenetic tree was constructed using the neighbor-joining (NJ) method basing on multiple alignments of sesame and *Arabidopsis COL* genes (Fig. 1a). The 13 *SiCOLs* were classified into three groups (I, II, and III) and each group consisted of 6, 3, and 4 SiCOL proteins, respectively. Two *SiCOL* genes, *SIN_1019889* and *SIN_1004896* showed the closest relationship with the *Arabidopsis CO* gene. The *Arabidopsis* CO protein sequence was also used as query for the BLAST to identify the homologous genes. It showed that *SIN_1019889* and *SIN_1004896* were the only homologous genes of *Arabidopsis CO* gene in sesame. Thus, these two genes were referred as *SiCOL1* (*SIN_1019889*) and *SiCOL2* (*SIN_1004896*), respectively. We therefore concluded that these genes might be involved in the photoperiodic regulation of sesame flowering.

Phylogenetic analysis of SiCOL1, SiCOL2, CO and CO homologous proteins in the other 19 plant species was performed. CO homologous proteins from monocots and dicots were clustered into two groups. Both SiCOL1 and SiCOL2 proteins were divided into the dicots group. SiCOL1 protein (GeneBank ID: XP_011085568) displayed the highest similarity to PnCO protein (the CO protein in *Pharbitis nil*, 53% identity, AF300700) whereas it showed a 44% identity with CO protein from *Arabidopsis* (NP_197088). SiCOL2 protein (XP_011099077) displayed the highest identity to SlCO protein (60% identity, NP_001233839) and StCO protein (60% identity, ARU77840), which was higher than that of *Arabidopsis* CO protein (48% identity). However, *SlCO* was not involved in the control of flowering time of *Solanum lycopersicum* [49]. Previous research suggested that sesame was taxonomically close to *Utricularia gibba*, *S. lycopersicum* and *S. tuberosum* [43]. However, in this study, *UgCO* protein was not close to either SiCOL1 or SiCOL2 protein.

### Conserved motifs and structure of the *SiCOL* genes

Using the *SiCOL* phylogenetic relationship data, we identified structural features of the sesame *COLs*, including conserved motifs and the locations of exons and introns (Additional file 1: Figure S1). The *SiCOL* genes of Group I and Group II had a simple gene structure -- one intron and two exons (Additional file 1: Figure S1b), while all genes in Group III had more exons and presented more complex gene structure than that of Group I and Group II. Multiple Em for Motif Elicitation (MEME) analysis confirmed the presence of the B-box motifs and CCT domains in *SiCOL* gene sequences. All genes in Group I and Group III had two B-box motifs except *SiCOL2*, which lacked one of the B-box motifs (Additional file 1: Figure S1c).

The protein sequences of *SiCOL1* and *SiCOL2* were further analyzed (Additional file 1: Figure S2). The result showed that they shared high similarity in amino acid sequence (61.7%), especially in the regions of B-box 2 motif (83.7%) and CCT domain (97.7%). SiCOL1 and SiCOL2 proteins had large differences in the B-box 1 motif region. Most amino acids of B-box 1 motif in SiCOL2 protein were lost. Even the remaining amino acids in B-box 1 motif of SiCOL2 protein were also quite different from that of *SiCOL1*. B-box motif plays an important role in the regulation of transcription and in mediating protein–protein interaction [50], and the missing of B-box 1 motif may cause loss of partial function of *SiCOL2*.

### Overexpression of *SiCOL1* and *SiCOL2* in *Arabidopsis*

**Fig. 2:**
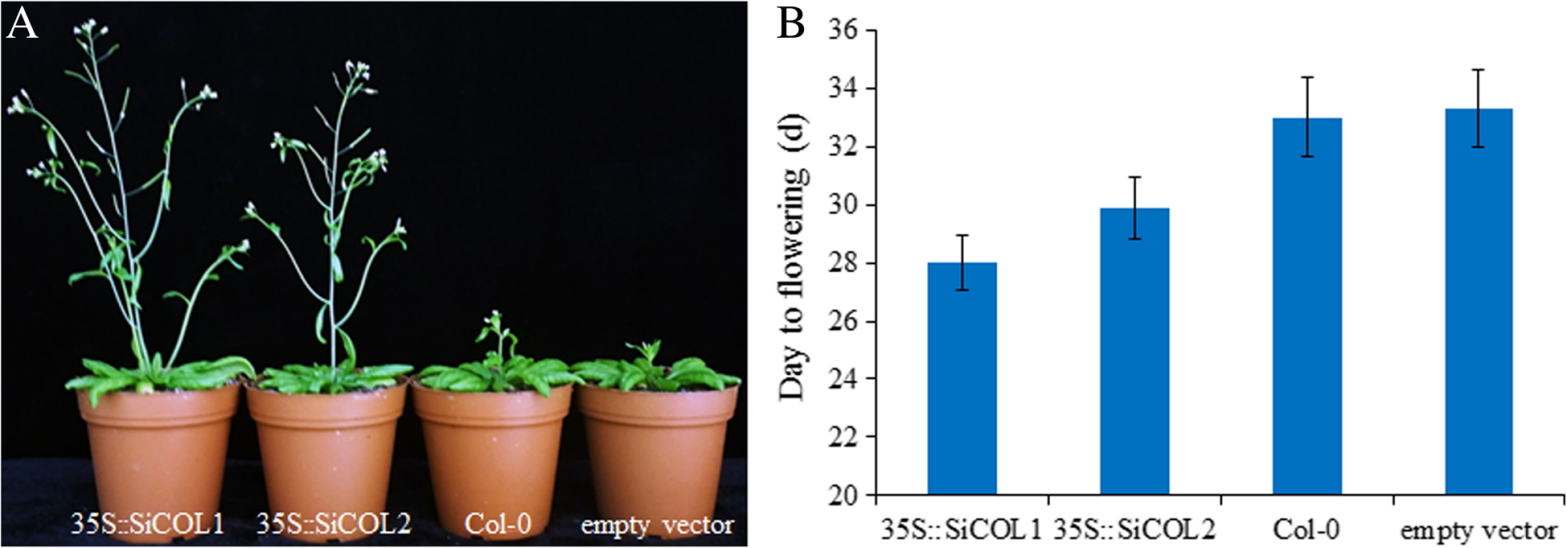
Days to flowering of transgenic *Arabidopsis* with overexpressed *SiCOL1* and *SiCOL2* under LD condition. **a** Flowering phenotype of T_2_ transgenic *Arabidopsis* lines with overexpressed *SiCOL1* and *SiCOL2*. Photo was taken at 7 d after flowering of the *SiCOL1* transgenic line. **b** Days to flowering of T_2_ transgenic *Arabidopsis* lines with overexpressed *SiCOL1* and *SiCOL2* under LD condition. The T_2_ transgenic *Arabidopsis* lines containing empty vector were used as control. For each test of *35S::SiCOL1*, *35S::SiCOL2* and empty vector, days to flowering of ten lines (each contained 10 plants) were counted (Additional file 1: Table S2). The bar indicates standard deviation

To explore the role of *SiCOL1* and *SiCOL2* in flowering, we constructed *SiCOL1* and *SiCOL2* overexpression vectors, and transferred into *Arabidopsis* Col-0 lines, respectively. Ten independent T_0_ transgenic lines were obtained for each gene. T_1_ generation transgenic lines planted in LD condition were about 3 days earlier flowering than the wild type. T_2_ generation plants were significantly earlier flowering (5 days of *35S::SiCOL1* on average, *P* < 0.001, and 3 days of *35S::SiCOL2* on average, *P* < 0.001) than the wild type (Fig. 2 and Additional file 1: Table S2). It is noteworthy that the T_2_ transgenic lines of *35S::SiCOL1* flowered earlier (2 days in average) than that of *35S::SiCOL2*. This result might be caused by the loss of B-box 1 motif in SiCOL2 protein. Therefore, we concluded that *SiCOL2* might lose partial function of flowering regulation and *SiCOL1* was potential functional homologous gene of *CO* in sesame.

To investigate the mechanism of action of *SiCOL1 and SiCOL2* in *Arabidopsis*, we compared the expression patterns of flowering related genes *FT* in transgenic lines with wild type under LDs. Under LDs, *FT* is induced by *CO* and promotes flowering in *Arabidopsis* [51]. Comparing with the *FT* in wild type, *FT* in the transgenic lines expressed in an extremely high level (Additional file 1: Figure S3). The result suggested that *SiCOL1* and *SiCOL2* promoted *Arabidopsis* flowering by inducing the expression of *FT*. Moreover, expression of *FT* in T_2_ transgenic lines with *35S::SiCOL1* was much higher than that in the *35S::SiCOL2* transgenic lines, indicating *SiCOL1* had higher induction efficiency of *FT* expression than *SiCOL2*.

### Expression patterns of *SiCOL1* and *SiCOL2*

**Fig. 3:**
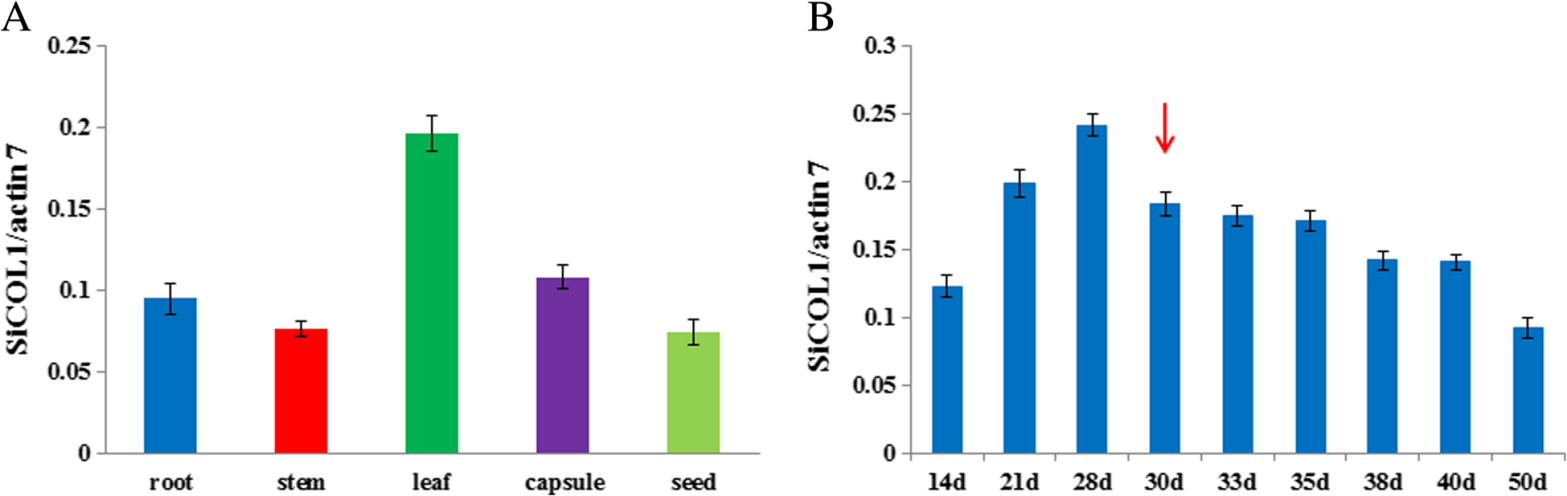
Relative expression of *SiCOL1* in different tissues and development stages of sesame. **a** Relative expression of *SiCOL1* in five tissues of sesame. **b** Relative expression of *SiCOL1* in leaves of different development stages. The red arrow indicates that tiny flower buds begin to appear in the axil of sesame plants. Transcript abundance was quantified using qRT-PCR and expression levels were normalized using sesame *actin7* as a reference gene. The bar indicates standard deviation

Five different tissues of sesame were collected from the widely cultivated sesame variety ‘Zhongzhi13’, including root, stem, leaf, capsule and seed. Quantitative real-time polymerase chain reaction (qRT–qPCR) was used to investigate the expression of *SiCOL1* and *SiCOL2* in these tissues. The result revealed that the expression of *SiCOL1* and *SiCOL2* in root, stem, capsule and seed were almost in the same level (Fig. 3a and Additional file 1: Figure S4a). However, both the expression levels of *SiCOL1* and *SiCOL2* in leaf were significantly higher than that in other tissues (*P* < 0.001).

Expression of *SiCOL1* and *SiCOL2* in leaf at the different development stages (from 14 days to 50 days after seed sowing) of ‘Zhongzhi13’ was investigated. All samples were collected in the same time (8:00 am) during a day. Generally, the flower buds of the variety ‘Zhongzhi13’ appear in approximately 30 days and ‘Zhongzhi13’ flowers at about 40 days in the growing season at Wuhan, China. The *SiCOL1* and *SiCOL2* expression increased quickly from 14 to 28 days and reached the highest level in 28 days, which was the exactly time before the flower buds appeared in the axil of sesame (Fig. 3b and Additional file 1: Figure S4b). After the flower bud appeared, the expression of *SiCOL1* moderately decreased (from 30 to 40 days). Although sesame is an indeterminate inflorescence species, the expression of *SiCOL1* decreased noticeably after the plant flowered (50 days). However, the expression of *SiCOL2* slightly increased after sesame flowering. The result suggested that the expression of *SiCOL1* and *SiCOL2* dynamic changed during the development of sesame floral organ.

**Fig. 4:**
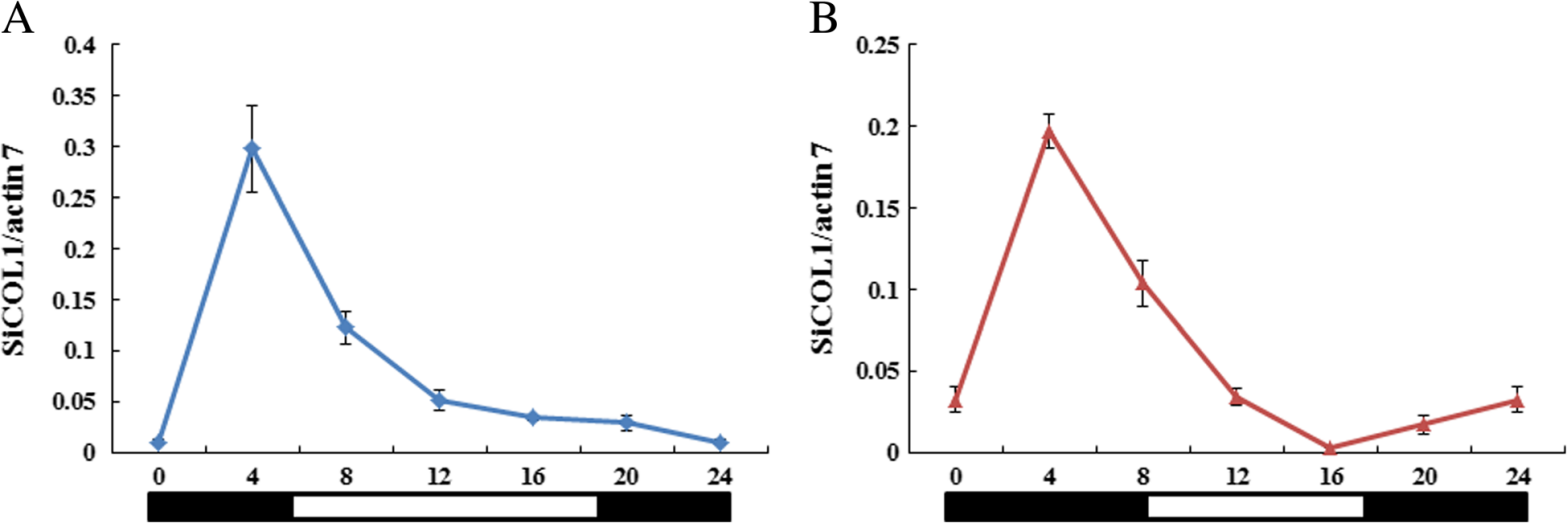
Relative diurnal expression of *SiCOL1* under LD and SD conditions. **a** Relative expression of *SiCOL1* under LD condition. **b** Relative expression of *SiCOL1* under SD condition. White boxes below the graphs indicate light periods and dark boxes indicate darkness. The expression data was normalized by sesame *actin7*. The bar indicates standard deviation

Individuals of ‘Zhongzhi13’ were grown in the LD (14 h light) and SD (9 h light) conditions, respectively. In about 3 days before the flower buds appeared, leaves from three individuals were collected during a 24 h period under LD and SD conditions, respectively. Expressions of *SiCOL1* and *SiCOL2* in the leaves under LD and SD conditions were detected. Although expression of *SiCOL2* was higher than *SiCOL1* in both LD and SD conditions, the expression patterns of these two genes were extremely similar. Both in LD and SD conditions, the expression of *SiCOL1* and *SiCOL2* increased during the darkness whereas decreased under light (Fig. 4 and Additional file 1: Figure S5). The peaks of transcript level of *SiCOL1* and *SiCOL2* in LD and SD conditions were both in the dawn. Under the SD condition, the lowest expression levels of *SiCOL1* and *SiCOL2* were both found at 1 h before dusk. Whereas, the valleys of the transcript levels for *SiCOL1* and *SiCOL2* under LD were different. Under LD condition, *SiCOL1* and *SiCOL2* had the lowest expression levels in 0 am and 8 pm, respectively. Therefore, as the homolog of *CO* in sesame, *SiCOL1* and *SiCOL2* exhibited significantly diurnal rhythmic expression and expressed in a high level before the flowering in leaves.

### Haplotype variation of *SiCOL1* and *SiCOL2*

In order to analyze the haplotype variations of *SiCOL1* and *SiCOL2*, SNPs of *SiCOL1* and *SiCOL2* in 132 landrace genomes were obtained from the SesameHapMap database (http://www.ncgr.ac.cn/SesameHapMap/). These landraces were collected from South Asia, Southeast Asia, East Asia and Central Asia. These regions are the main producing regions of sesame with rich germplasm resources. Among these regions, South Asia is also the geographic origin area of sesame [9, 52]. All samples were planted in the summer of Wuhan, China from 2015 to 2017 and their flowering dates were recorded. Previous study revealed that sesame accessions could be divided into south group and north group by the latitude 32°N [13]. In the present study, samples were also divided into south and north groups according to their geographic origin (Additional file 1: Table S3).

**Fig. 5:**
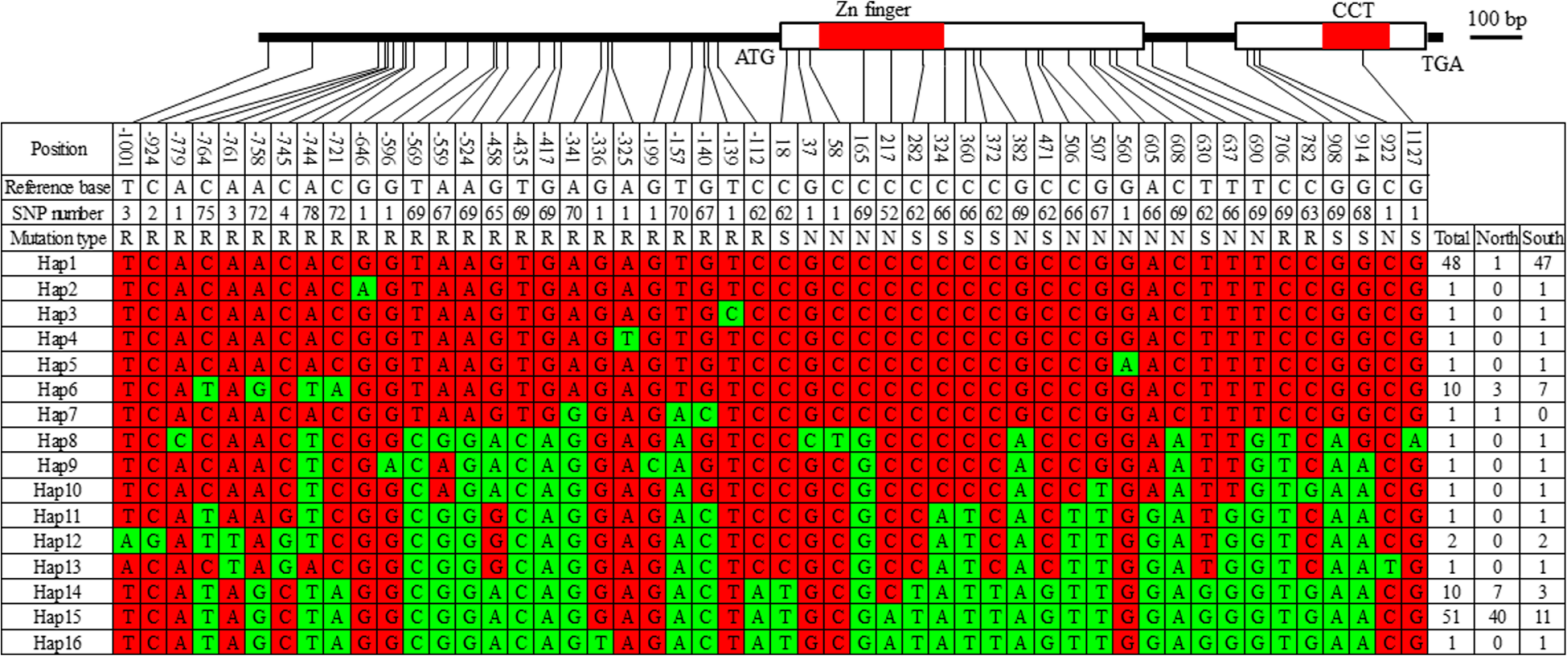
Haplotypes of *SiCOL1* among landraces from Asia. Reference base is the base in reference genome ‘Zhongzhi13’. SNP number is the mutation number among the 132 landraces. R, S and N in mutation type indicate replacement, synonymous SNP and nonsynonymous SNP, respectively. Numbers in the right column are numbers of cultivars represented in every haplotypes. Total, South and North indicate total landraces, landraces from south group and landraces from north group, respectively. Variations that different from the reference bases are shown in green

In total, 25, 23 and 2 SNPs were found in the promoter, coding region and intron of *SiCOL1*, respectively (Fig. 5). Among the 23 SNPs in the coding region, 13 SNPs were the synonymous mutations while the other 10 SNPs were the nonsynonymous mutations, which led to amino acid substitutions and might cause functional polymorphism of the SiCOL1 protein. Only one SNP and three SNPs were detected in the CCT domain and Zinc-finger domain, respectively.

Based on the identified SNPs, 16 haplotypes of *SiCOL1* were detected in the tested sesame accessions. All bases in Hap1 (Haplotype 1) were the same as the reference genome ‘Zhongzhi13’ [43]. The bases in Hap1 that were different from other haplotypes ranged from 1 to 35. Six of the haplotypes (Hap2 to Hap7) were similar to Hap1 while the other nine haplotypes (Hap8 to Hap16) were quite different from Hap1. There was only one SNP in Hap 2, Hap3, Hap4 and Hap5. But in Hap 14, Hap 15 and Hap16, the different bases reached 33, 34 and 35, respectively.

The variety ‘Baizhima’ (S054 in Additional file 1: Table S3), which had the *SiCOL1* of Hap15 was selected and the expression of *SiCOL1* and *SiCOL2* was investigated. *SiCOL2* showed diurnal rhythmic expression in ‘Baizhima’ under both LD and SD conditions (Additional file 1: Figure S5). However, the expression of *SiCOL1* was not detected in ‘Baizhima’ under both LD and SD conditions, suggesting that mutated *SiCOL1* did not express and might lose the function of photoperiod response in sesame flowering.

Totally, 15 SNPs were identified in *SiCOL2*, including seven SNPs in promoter, six SNPs in coding regions and two SNPs in intron (Additional file 1: Figure S6). Four SNPs in the coding regions were the nonsynonymous mutations. However, these SNPs were identified in a few samples, indicating that *SiCOL2* was more conserved than *SiCOL1*. Using the 15 SNPs, *SiCOL2* was clustered into 12 haplotypes. The haplotypes contained more than 7 accessions (5.30% of the total samples) were regarded as major haplotypes. Therefore, Hap1, Hap3 and Hap8 were identified to be the three major haplotypes. Among these haplotypes, Hap1 was the biggest haplotype, containing 65.2% of the total samples.

To valid the truth of the SNPs in *SiCOL1* and *SiCOL2*, ten accessions were selected and sequenced. All SNPs identified in *SiCOL1* and *SiCOL2* of the ten samples were the same as them in SesameHapMap. The result suggested that all SNPs of these genes were true and could be used in the haplotype analysis. However, a 6 bp deletion (from 421 bp to 426 bp) in the coding region, which resulted in an Aspartic acid and a Glutamic acid deletion in protein, was detected in Hap15 of *SiCOL1* (Additional file 1: Figure S7). Previous study showed that a 36 bp deletion in the coding region of *Hd1* was the crucial mutation that led function divergence of *Hd1* in rice [2]. This deletion might have potential influence of gene function in the Hap15 of *SiCOL1*.

**Fig. 6:**
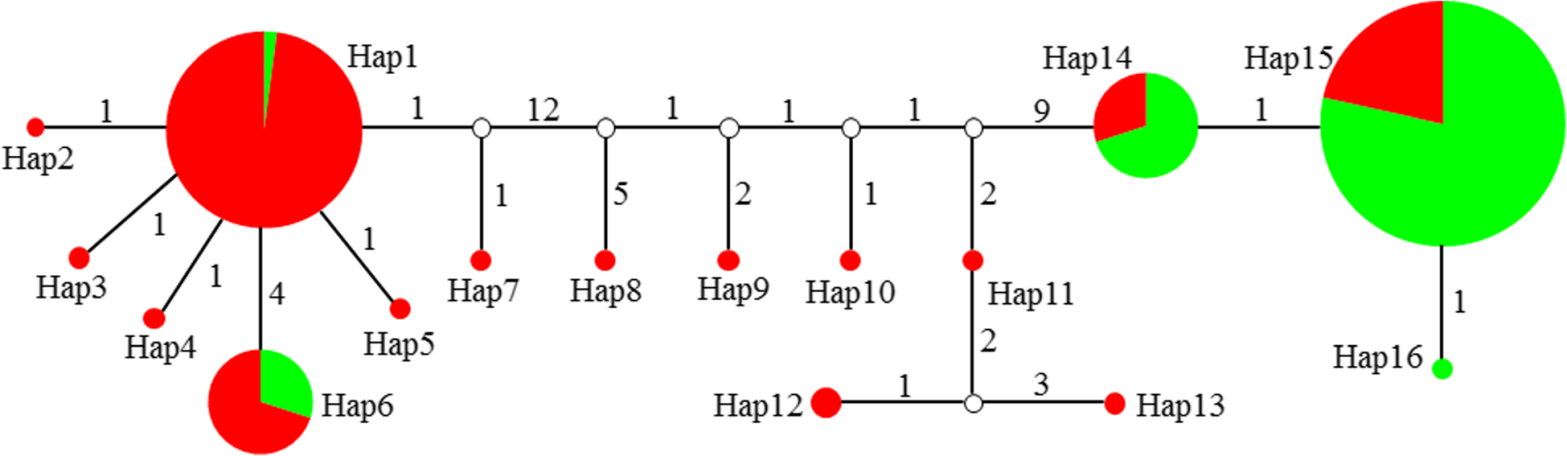
Haplotype network of *SiCOL1*. Haplotypes are showed by colored solid circles. Circle size is proportional to the quantity of samples within a given haplotype. Hollow circles indicate the assumed haplotypes. Lines between haplotypes represent mutational steps between alleles. The numbers next to the lines indicate the nucleotide difference existed between the linked haplotypes. The red color and green color indicate landraces from south group and north group, respectively

As shown in Fig. 6, a network of all haplotypes was constructed. The haplotype number of landraces from south group (15) was much more than that of north group (5), suggesting that *SiCOL1* had highly polymorphisms in the landraces of south group. There were four haplotypes contained landraces from both south and north group: Hap1, Hap6, Hap14 and Hap15. These four haplotypes were also the largest haplotypes in number, containing 90.2% (119 of 132 landraces) of the samples. The landraces belonging to south group were concentrated in Hap 1 and Hap6 (54 of 80 landraces), while most of the landraces from north group were in Hap14 and Hap15 (47 of 52 landraces).

The landraces from India presented in Hap1, Hap5, Hap6, Hap8, Hap9, Hap11, Hap12 and Hap13, indicating a high genetic diversity of *SiCOL1* in India sesame landraces. If we take all landraces from South Asia (India, Bangladesh, Pakistan and Nepal) into account, more haplotypes could be found, including Hap4, Hap7, Hap10, Hap 15 and Hap16. Therefore, landraces from South Asia could be found in 13 haplotypes totally. For Southeast Asia, East Asia and Central Asia, the haplotypes of landraces from these regions were Hap7, Hap5 and Hap2, respectively. The haplotypes of landraces from South Asia were much more than haplotypes including landraces from other regions, suggesting that South Asia was the genetic diversity center of *SiCOL1*. This observation is consistent with previous suggestion that crop cultivars from the geographic origin areas tend to have higher genetic diversity [53, 54].

A network of all *SiCOL2* haplotypes was also constructed (Additional file 1: Figure S8). Landraces from south group and north group were detected in twelve and five haplotypes, respectively. In the network of *SiCOL1*, two major haplotypes, Hap14 and Hap15 were dominated by the landraces from north group. However, landraces from south group were more than that from north group in all major haplotypes of *SiCOL2* (Hap1, Hap3 and Hap8).

### *SiCOL1* haplotypes were related to sesame flowering

**Fig. 7:**
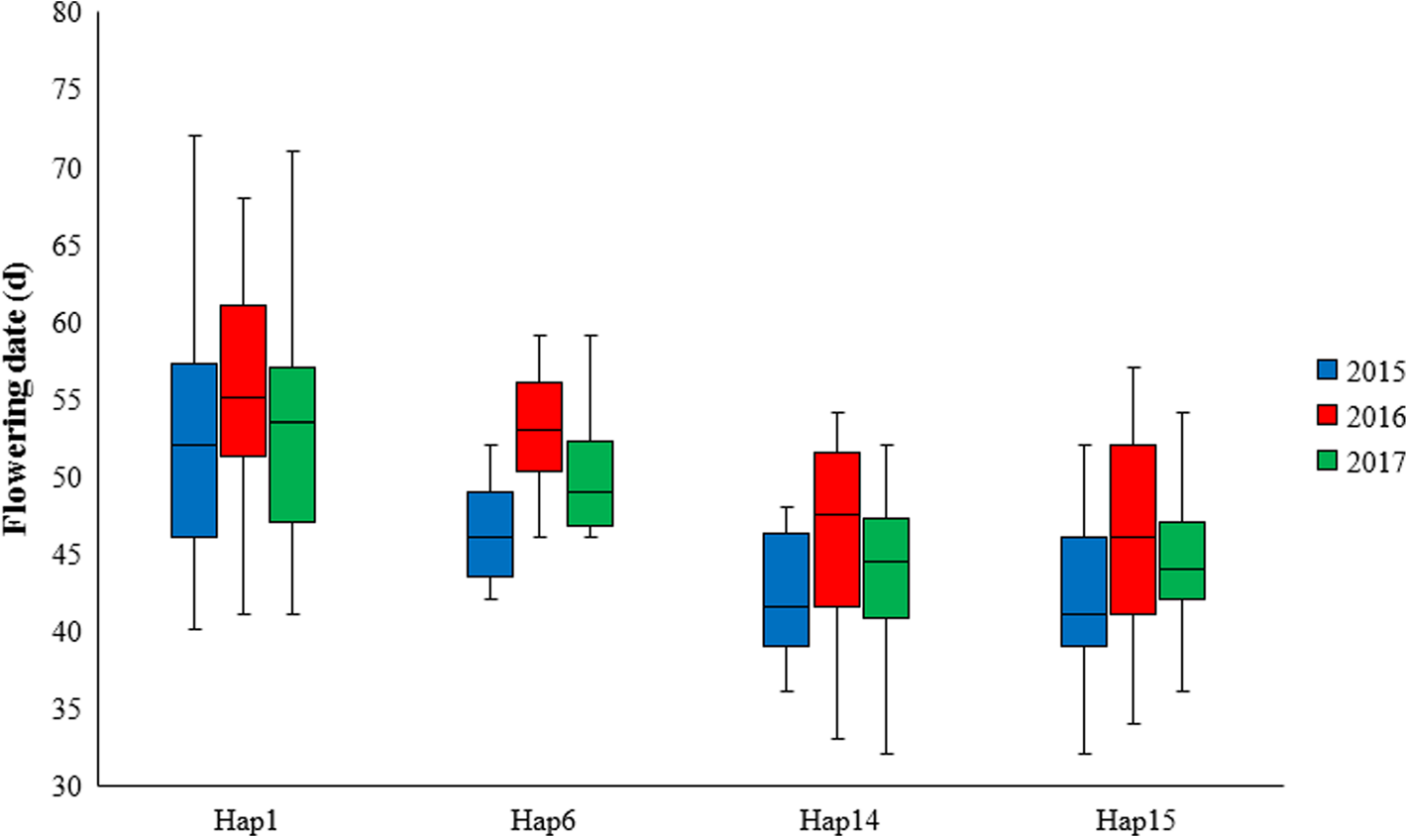
Box-plot of the flowering date of sesame landraces in major haplotypes. The major haplotypes, Hap1, Hap6 Hap14 and Hap15 contained 48, 10, 10 and 51 sesame accessions, respectively. All sesame landraces were planted from May to October at Wuhan, China in every year. The detailed information of the flowering date of the sesame landraces was provided in Additional file 1: Table S3

The flowering date of the 132 landraces from 2015 to 2017 in Wuhan, China (114°33′ E, 30°34′ N) was recorded and analyzed to further examine the relationship between *SiCOL1* haplotypes and sesame flowering (Additional file 1: Table S3). The day light in the summer of Wuhan is a standard LD, sustaining from 13 h to 14.5 h. Under LDs, sesame landraces from north group flowering obviously earlier than that from south group. The box-plot showed the flowering date of landraces in Hap1, Hap 6, Hap14 and Hap15 from 2015 to 2017 (Fig. 7). As we described previously, Hap1 and Hap6 mainly contained sesame accessions from south group, while Hap14 and Hap15 included most sesame accessions from north group. Days to flowering time of the samples in Hap1 and Hap6 were significant more than that in Hap14 and Hap15 (Mann-Whitney test, *P* < 10^− 9^). Taking flowering time in 2016 for example, the average flowering date of accessions in Hap1, Hap6, Hap14 and Hap15 was 58.5, 53, 46.2 and 46.3 d, respectively. The Pearson correlation coefficient was used to test the correlation between *SiCOL1* haplotypes and flowering date. Significant correlations were identified in all 3 years: 2015 (*R*^2^ = 0.32, *R* = 0.56, *P* = 3.10 × 10^− 11^), 2016 (*R*^2^ = 0.28, *R* = 0.53, *P* = 5.38 × 10^− 10^) and 2017 (*R*^2^ = 0.30, *R* = 0.55, *P* = 7.80 × 10^− 11^). The results suggested that *SiCOL1* variations were strongly related to the flowering time of sesame.

### Geographic distribution of *SiCOL1* haplotype

**Fig. 8:**
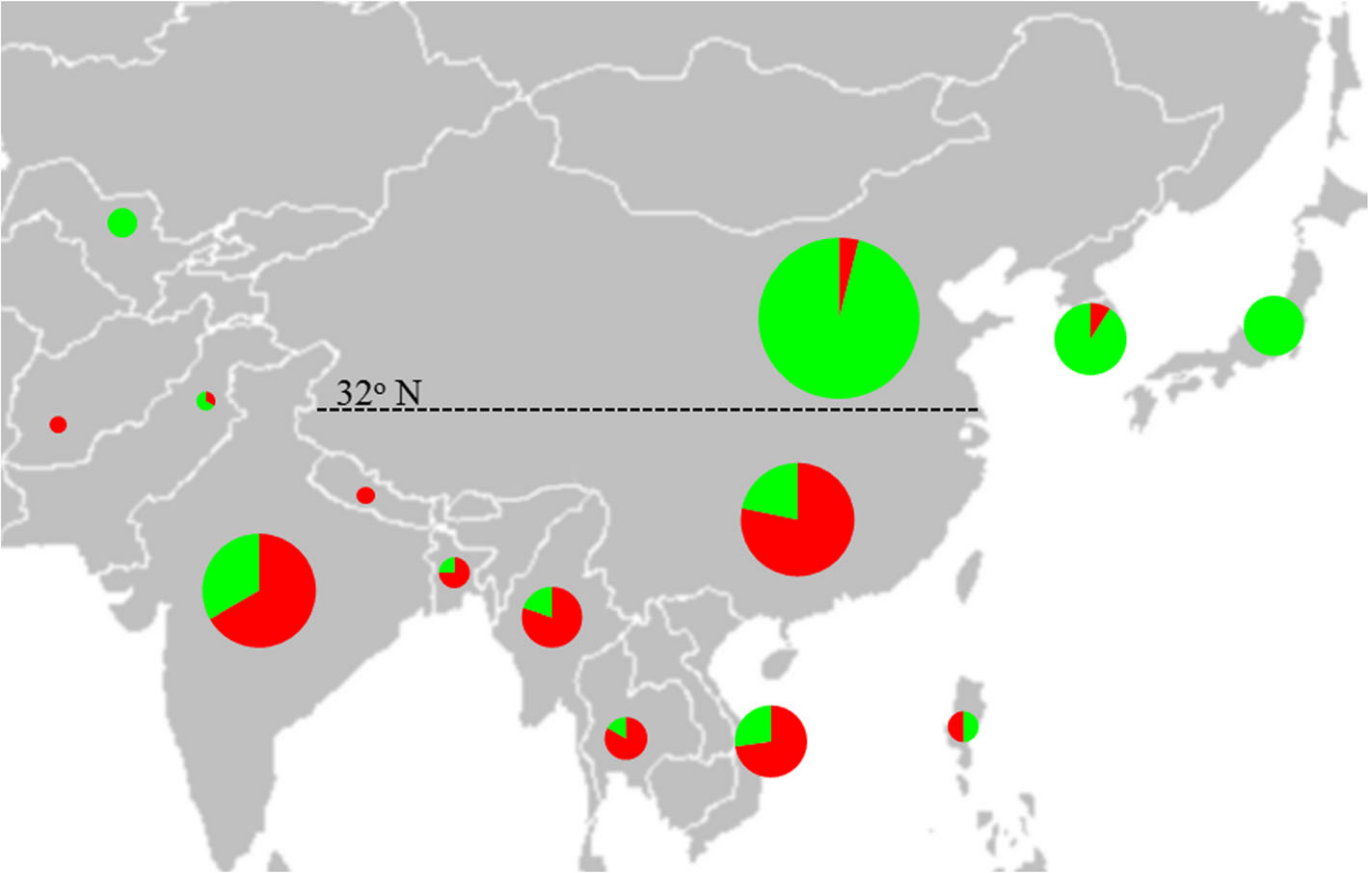
SiCOL1 protein type distribution among countries in Asia. Red solid circles indicate SiCOL1 protein types from Hap1 to Hap7, while the green solid circles represent SiCOL1 protein types from Hap8 to Hap16. The size of the circles is proportional to the quantity of sesame landraces. The latitude 32°N is indicated by dotted line. The original map was downloaded and adapted from “https://commons.wikimedia.org/wiki/File:BlankMap-Asia.png“(Bytebear at the English language Wikipedia). This original map is licensed under the Creative Commons Attribution-Share Alike 3.0 Unported license, which allows us to share and adapt for free with proper attribution

Comparing to Hap1 of *SiCOL1*, Hap15 had one 6 bp deletion in the coding region (Additional file 1: Figure S7) and many SNPs in the promoter as well as coding regions (Fig. 5). In addition, Hap15 did not express under both LD and SD conditions. Therefore, Hap15 of *SiCOL1* was regarded as nonfunctional allele. Based on the similarity of haplotypes, we divided the 16 haplotypes of *SiCOL1* into two groups, south haplotypes with functional alleles and north haplotypes with nonfunctional alleles. The south haplotypes included Hap1 to Hap7 while the north haplotypes contained Hap 8 to Hap 16. To investigate the relationship between the geographic origin and haplotypes of the sesame landraces, a map of Asia was downloaded from Wikimedia Commons (http://commons.wikimedia.org/wiki/Main_Page) and the distribution information of *SiCOL1* haplotypes was showed in the map (Fig. 8). The map clearly showed that south haplotypes mainly existed in the south of 32°N while north haplotypes were concentrated in the north of 32°N. For the 13 countries, the proportion of the north haplotypes ranged from 0 (Nepal and Afghanistan) to 100% (Japan and Uzbekistan).

Since alleles contained in north haplotypes all were nonfunctional and very few landraces in the north haplotypes were from the geographic origin center of sesame, north haplotypes were regarded as the domesticated haplotypes of *SiCOL1*. The frequency of domesticated alleles is an indicator of artificial selection, so the proportion of the north haplotypes was used to examine the domestication and spread of sesame. North haplotypes were in the minority of Southern Asia, Southeast Asia and South China, but they were the dominant haplotypes in Northern China, Northeast Asia and Central Asia. Therefore, the result suggested that *SiCOL1* had been strongly selected and might be the important domesticated gene that contributed to the spread of sesame from low-latitude regions to high-latitude regions.

### Expression patterns of *SiFT* in two varieties with different *SiCOL1* haplotypes

**Fig. 9:**
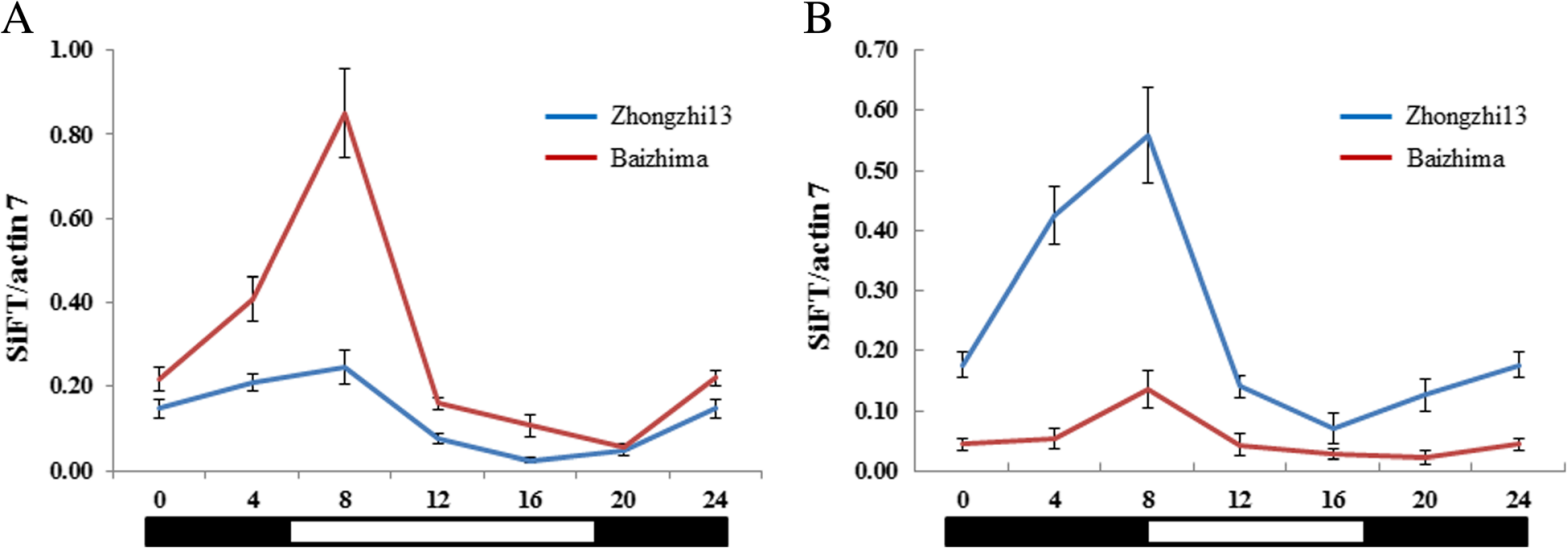
Relative diurnal expression of *SiFT* under LD and SD conditions. **a** Relative expression of *SiFT* under LD condition. **b** Relative expression of *SiFT* under SD condition. White boxes below the graphs indicate light periods and dark boxes indicate darkness. The expression data was normalized by sesame *actin7*. The bar indicates standard deviation

The homolog of *FT* in sesame, *SiFT* (*SIN_1009320*), was identified by BLAST [55]. Expression of *SiFT* was detected in ‘Zhongzhi13’ (with *SiCOL1* of Hap1) and ‘Baizhima’ (with *SiCOL1* of Hap15) under LD and SD conditions. The diurnal rhythmic expression pattern of *SiFT* was quite similar to that of *SiCOL1* under both LD and SD conditions (Fig. 9), indicating that the expression of *SiFT* might be induced by *SiCOL1*. Although the expression pattern of *SiFT* in ‘Zhongzhi13’ and ‘Baizhima’ was similar, the expression level of *SiFT* in these two varieties was quite different under both LD and SD conditions. These significantly variant expression levels of *SiFT* in ‘Zhongzhi13’ and ‘Baizhima’ might result from the non-expression of *SiCOL1* in ‘Baizhima’.

The peak of *SiFT* expression appeared later than that of *SiCOL1*. This phenomenon was in line with the homologue genes, *Hd3a* and *Hd1*, in the SD plant rice. Although *Hd1* had the expression peak in dark, *Hd3a* had the highest expression level after dawn under both LD and SD conditions [56].

## Discussion

### *SiCOL1* might be involved in the photoperiod response and contributing to flowering

Photoperiod pathway is one of the crucial regulation factors of high plant flowering [57]. *CO*, one of the first identified plant photoperiod gene, plays an important role in the photoperiod response and flowering regulation of *Arabidopsis* [3]. The *CO* homolog of sesame has not been identified and the flowering mechanism of sesame largely remains unknown. In this study, molecular function, gene expression and sequence variations of the *CO* homolog in sesame, *SiCOL1*, were comprehensively analyzed.

Phylogenetic analysis showed *SiCOL1* was one of the most similar genes of *CO* in sesame. Overexpression of *SiCOL1* in the transgenic *Arabidopsis* lines significantly promoted flowering of *Arabidopsis* under LD condition. Both under LD and SD conditions, *SiCOL1* showed diurnal rhythmic expression and had the peak expression at the dawn. Compared with the *SiCOL1* expression in root, stem, capsule and seed, *SiCOL1* had a higher expression level in leaf. The expression pattern of *SiCOL1* was extremely similar to that of *AtCO1* and *AtCOL2* [58]. Although *AtCOL1* and *AtCOL2* doesn’t have major role in the control of flowering in *Arabidopsis*, homologous genes with similar dawn expression peaks have been shown to control *FT* expression and flowering time in other species including soybean and strawberry [59, 60]. The diurnal rhythmic expression pattern of *SiFT* was similar to that of *SiCOL1*, indicating that *SiCOL1* might induce the expression of *SiFT*. In ‘Zhongzhi13’, *SiFT* had a higher expression level under SD condition than that under LD condition. It was consistent with the early flowering of ‘Zhongzhi13’ under SD condition. By analyzing the days to flowering of 132 landraces, we found that the north haplotypes of *SiCOL1* harboring nonfunctional alleles flowered much earlier than that of other landraces under LD condition. In the variety ‘Baizhima’, which had Hap15 of *SiCOL1*, *SiFT* had a high expression level under LD condition. Therefore, the early flowering of sesame landraces, which had the nonfunctional haplotypes of *SiCOL1*, might result from the highly expression of *SiFT* under LD conditions. Since *SiCOL1* did not express in these landraces, we concluded that *SiCOL1* might repressed the expression of *SiFT* under LD condition. *Hd1* functions in the promotion of rice flowering under SD condition and in inhibition under LD condition [2]. We speculated that *SiCOL1* might have similar function of *Hd1* in the photoperiod response and contributing to flowering. Because the transgenic approach of sesame had not been invented, it is hard to perform the transgenic experiments to validate the function of *SiCOL1* in sesame. This will be addressed in future studies.

Besides *SiCOL1*, there were 12 other *SiCOL* genes that were identified from sesame genome. Function of these genes had not been reported yet. In the last decades, *COL* gene family has been studied in several plants [22, 23, 30, 36–39, 41]. In *Arabidopsis*, 17 COL genes were identified totally. Due to the differences of B-box motifs and introns, the *AtCOLs* were divided into three groups [41], Group I contained *CO* and *COL1* to *COL5* with two B-box motifs and one intron. Group II includes *COL6* to *COL8* and *COL16* with one B-box motif and one intron. Group III incorporates *COL9* to *COL15* with two B-box motifs and three introns [23]. Similar result had been recognized in sesame *COL* genes. However, *SiCOL2* which belonged to Group I lacked B-box 1 motif, indicating the possible divergence of *SiCOLs* and *AtCOLs*. Previous studies showed that *Arabidopsis COL* genes not only regulated flowering time, but also participated in plant architecture, development, and stresses tolerance [61–64]. The *SiCOLs* might be also involved in diverse molecular and genetic processes of sesame.

### *SiCOL1* rather than *SiCOL2* is more likely to be the functional homologous gene of *CO* in sesame

Phylogenetic analysis of *SiCOL1*, *SiCOL2*, *CO* and *CO* homologues of 19 plant species showed *SiCOL1* was close to *PnCO*, whereas *SiCOL2* was close to *SlCO* and *StCO*. Previous study showed that *PnCO* could promote flowering of *P. nil* [25], a typical SD plant. However, there is no evidence shows that SlCO can regulate flowering of *Solanum lycopersicum* [49], a day-neutral plant. Comparison of *SiCOL1* and *SiCOL2* protein sequences and motifs revealed differences in the Zinc-finger domain that could be the underlying reason for differences in function. When we overexpressed *SiCOL1* and *SiCOL2* into *Arabidopsis*, the flowering time promoted by *SiCOL2* was less than *SiCOL1*. In addition, the expression pattern of *SiCOL2* was different from that of *SiFT* in the variety ‘Baizhima’ (Additional file 1: Figure S5). These results suggested that *SiCOL1* rather than *SiCOL2* was more likely to be the functional *CO* homologous gene in sesame.

Much fewer SNPs were detected in the coding region (6 SNPs) and domains (2 SNPs) of *SiCOL2* than that of *SiCOL1* (Additional file 1: Figure S6). Two SNPs were detected in the B-box motif and CCT domain of *SiCOL2*. But only one was nonsynonymous SNP and few of the landraces contained this mutation in *SiCOL2*. Totally, there were 12 haplotypes of *SiCOL2* and most sesame accessions were concentrated in Hap1, Hap3, Hap5 and Hap8 (Additional file 1: Figure S8). The *SiCOL2* haplotype network showed that the major haplotypes had an extremely close relationship and landraces from south and north group were mixed in the haplotypes. The results indicated that variations of *SiCOL2* might not affect the flowering of sesame and *SiCOL2* had not been significantly selected.

Genome research uncovered an independent whole genome duplication (WGD) event in sesame genome at approximately 71 ± 19 million years ago [43]. The paralogs, *SiCOL1* and *SiCOL2*, may be the duplicated genes. Functional divergence of these paralogs might result from the loss of B-box 1 motif in *SiCOL2*. Redundant genes resulting from WGD are thought to be lost or acquire new functions [65]. *SiCOL2* might lose its gene function after WGD.

### Artificial selection of *SiCOL1* might have contributed to sesame spread to a wide range of latitudes

Gene sequence analysis of *SiCOL1* revealed that two nonsynonymous mutations which caused amino acid residues replacement were in the Zinc-finger domain. In addition, one 6 bp deletion in the coding region was detected in the haplotypes harboring this mutation. The amino acid residues replacement in Zinc-finger domain, the 6 bp deletion, and multiple SNPs in the coding regions as well as promoter might result in the loss of function of *SiCOL1*. Landraces which contained these mutations mainly distributed in high-latitude regions and flowered early in LD conditions. In contrast, most landraces from low-latitude regions, especially South Asia, which was the domestication center of sesame, did not have these mutations and flowered late in LD conditions. Photoperiod genes in the wild relatives of crops, such as rice, maize and soybean are generally functional and photoperiod genes tend to be selected during the crop spread [66–68]. Therefore, the functional *SiCOL1* in the samples from South Asia was more likely to be the ancestral haplotypes. Further haplotype analysis of *SiCOL1* in the Asia sesame collections revealed that the landraces from north group containing nonfunctional *SiCOL1* alleles distributed across Northern China, Northeast Asia and Central Asia. Northeast Asia is in the northern-limit regions of sesame, with more than 15 h mean day length during the short growing season. Almost all sesame landraces in Northeast Asia had a few haplotypes with nonfunctional mutations and flowered early under LD condition. The haplotypes of landraces from Northeast Asia was significantly less than that from South Asia. Additionally, the landraces from Northeast Asia harbored nonfunctional haplotypes of *SiCOL1*. The results suggested *SiCOL1* in the landraces from Northeast Asia might undergo positive selection or strong domestication and *SiCOL1* played a significant role in sesame adaptation to high-latitude regions by reducing photoperiod sensitivity. Domestication and selection on *SiCOL1* might be one of the critical events that contribute to adapt sesame to different cultivation areas and cropping seasons, resulting in sesame from a local crop in India to the global oilseed crop.

Several studies of the rice photoperiod genes have reported that selection of the flowering genes mainly contributed to the expansion of rice from tropical and subtropical areas to temperate areas, resulting in rice changing from a regional plant to a worldwide plant [33, 69–71]. Domestication of the photoperiod genes, such as *Hd1*, *Ehd1*, *Hd3a*, *Ghd7*, *Ghd8*, and *DTH2*, caused function loss and decreased the photoperiod sensitivity, leading to early-heading phenotypes. The artificial selection and domestication of rice flowering genes successfully extended the northern-limit regions of rice cultivation. In this study, similar phenomenon was observed in the sesame photoperiod gene *SiCOL1*. Artificial selection and domestication of *SiCOL1* might contribute the early flowering of nonfunctional haplotypes and involved in the spread of sesame from low-latitude area (South Asia) to high-latitude areas (Northeast Asia and Central Asia). Since the function of different haplotypes had not been completely demonstrated by the sesame transformation approach, and there was weak population structure in the sesame landraces [13], this conclusion still need more evidences to support.

To date, more than 700 quantitative trait loci (QTLs) and 30 photoperiod genes had been identified in rice [72]. Natural variations that related to rice flowering were found in 14 genes. In the present study, we found that some extremely early-heading landraces contained nonfunctional alleles of *SiCOL1*. For example, landraces containing the *SiCOL1* of a nonfunctional haplotype (Hap14) flowers at 43.6 d in average under LD condition. But the accession ‘Baizhima’ (S049 in Additional file 1: Table S3) from Northeast China (125°8′ E, 45°51′ N), which also harboring *SiCOL1* of Hap14, flowers quite earlier (30.7 ± 1.3 d) than other accessions. These finding suggested that sesame domestication in the northernmost regions might have been achieved by artificial selection of *SiCOL1*, as well as domestication in other photoperiod genes. Furthermore experiments needed to be carried out to recognize these photoperiod genes in sesame.

*SiCOL1* identified in this research could be used in the sesame improvement and molecular breeding. Because of the simple and efficient of sesame artificial hybridization, any favorable allele of *SiCOL1* in landraces can easily be transferred to commercial varieties for adapting to different light conditions. The gene editing technology, CRISPR/Cas9, had been successfully used in the editing of tomato flowering gene *SELF PRUNING 5G*, causing 2 weeks earlier flowering [73]. Using the CRISPR/Cas9 method on editing of photoperiod genes, such as *SiCOL1*, geographical range of sesame could be extended. Sesame might be grown in latitudes more northerly than currently possible, which could also bring more plantings per growing season and thus higher yield of sesame.

## Conclusions

Flowering and photoperiod sensitivity are fundamental traits that determine sesame, an important oilseed crop, adaptation to a wide range of geographic environments. Whereas the flowering mechanism of sesame is still not clear. In the present study, we identified sesame *COL* gene family and focused on functional analysis of the *CO* homologous gene, *SiCOL1*. Phylogenetic analysis and sequence comparison revealed that *SiCOL1* might be the homolog of the *CO* gene in sesame. Overexpression of the *SiCOL1* in transgenic *Arabidopsis* significantly promoted flowering of *Arabidopsis* under LD conditions. Expression analysis revealed that *SiCOL1* had highly expressed levels in leaf before flowering and exhibited a diurnal rhythmic expression under both SD and LD conditions. Moreover, *SiCOL1* might induce the expression of *SiFT* under both SD and LD conditions. In the Asia sesame collections, different haplotype alleles of *SiCOL1* were found. However, the mutated haplotype (Hap15) of *SiCOL1* did not express under both SD and LD conditions. The similar haplotypes of Hap15 were regarded as nonfunctional alleles of *SiCOL1*. Notably, the sesame varieties from high-latitude regions harboring nonfunctional alleles of *SiCOL1* flowered extremely early, and were adapted to the northernmost regions of sesame cultivation. The results suggested that *SiCOL1* was the potential functional homolog of *CO* and haplotype variations of *SiCOL1* enables sesame to adapt to different day-lengths characteristic of different latitudes. Moreover, the domestication and artificial selection of *SiCOL1* might have contributed to the spread of sesame from low-latitude regions to high-latitude regions. Our results could be useful in both understanding the flowering mechanism and the molecular breeding of sesame.

## Methods

### Identification of the *COL* gene family in sesame

All sesame protein sequences were obtained from the sesame genome database (http://ocri-genomics.org/Sinbase/) [74]. The *Arabidopsis thaliana AtCOL* gene sequences were downloaded from TAIR (https://www.arabidopsis.org/). The HMM profile for the Zinc-finger B-box domain (PF00643) and CCT domain (PF06203) were downloaded from the PFAM protein families database (http://pfam.xfam.org) [75] and used to identify *COL* genes from the sesame genome with HMMER 3.0 [76]. BLAST analysis with all the *Arabidopsis COLs* was used to check the predicted *COLs* from the sesame database [55]. The CDD (http://www.ncbi.nlm.nih.gov/cdd/) [77] and the simple modular architecture research tool (SMART) [78] were used to validate all the potential sesame *COL* genes identified by HMM and BLAST if they contained the B-box motifs and CCT domains.

### Phylogenetic and sequence analyses of the *COL* gene family in sesame

Clustal X 2.0 [79] was used to align the aa sequences of the sesame and *Arabidopsis* COL proteins. A unrooted NJ phylogenetic tree [80] of these genes was constructed by MEGA 6.0 [81]. The nodes of the NJ tree were evaluated by bootstrap analysis for 1000 replicates. Branches with less than 50% bootstrap values were collapsed.

Twenty eight protein sequences of *CO* and *Hd1* homologs in plant species were download from NCBI, including *AtCO* (*A. thaliana*, X94937), *AtCOL1* (AED92215), *AtCOL2* (AEE73800), *BnCO* (*Brassica napus*, AY290868), *BdHd1* (*Brachypodium distachyon*, XP_003563958), *GhCOL1-A* (*Gossypium hirsutum*, ASA69414), *GhCOL1*-*D* (ASA69421), *GmCOL1a* (*Glycine max*, NP_001235828/ Glyma.08G255200.1, the gene ID in *G. max* genome Wm82.a2.v1), *GmCOL1b* (NP_001235843/Glyma.18G278100.1), *GmCOL2a* (XP_003541197/Glyma.13G050300.1), *GmCOL2b* (NP_001278944/Glyma.19G039000.1), *HvCO1* (*Hordeum vulgare*, AF490468), *LtCO* (*Lolium temulentum*, AY553297), *MdCOL1* (*Malus domestica*, AAC99309), *OsHd1* (*Oryza sativa*, AB041838), *PdCOL1* (*Populus deltoids*, AAS00054), *PdCOL2* (AAS00055), *PnCO* (*P. nil*, AF300700), *PpCOL1* (*Physcomitrella patens*, BAD89084), *PrCO* (*Pinus radiate*, AF001136), *RsCOL1* (*Raphanus sativus*, AF052690), *SiCOL1* (*S. indicum*, XP_011085568), *SiCOL2* (*S. indicum*, XP_011099077), *SlCO* (*S. lycopersicum*, NP_001233839), *StCO* (*S. tuberosum*, ARU77840), *TaHd1* (*Triticum aestivum*, AB094490), *VvCO* (*Vitis vinifera*, CBI16899), *ZmHd1* (*Zea mays*, ABW82153). Another *CO* homolog, *UgCO* (Scf02496.g25887.t1) was identified by BLAST with *AtCO* from the genome of *U. gibba* [82], which had taxonomically close relationship with sesame. Phylogenetic tree of *CO* and 21 *CO* homologs, including *SiCOL1* and *SiCOL2*, was constructed by NJ method, with the 1000 replications bootstrap analysis.

The conserved motifs in the full-length COL proteins were identified using the MEME program (http://alternate.meme-suite.org/tools/meme) [83]. The parameters employed in the analysis were as follows: maximum number of motifs = 3; optimum width of motifs = 15–60. The exon/intron structures of the *SiCOL* genes were determined by comparing their predicted coding sequence (CDS) with genomic sequences using the gene structure display server web-based bioinformatics tool (http://gsds.cbi.pku.edu.cn/) [84].

### Plant samples and treatments

The photoperiod-sensitive sesame variety ‘Zhongzhi13’ was selected and used for the gene expression analysis. It is widely cultivated in China and has been used in the genome sequencing of the sesame [43]. The materials were planted in summer of 2015 at Wuhan, China (114°33′ E, 30°34′ N). At the flowering stage, the roots, stems, leaves, capsules and developing seeds were collected from three plants of the variety ‘Zhongzhi13’ from 8:00 to 9:00 am during the day. After collection, these organs were immediately frozen in liquid nitrogen and stored at − 80 °C prior to further analysis. Leaves at 10 development stage of the variety ‘Zhognzhi13’ were collected, including 7, 14, 21, 28, 30, 32, 36, 38, 40 and 50 d. These leaves were collected at 8:00 am every time using the described method previously.

For the LD and SD treatments, sesame plants of ‘Zhongzhi13’ and ‘Baizhima’ (S054 in Additional file 1: Table S3) were firstly planted in pots at natural light condition for 1 week. Then the LD and SD treatment plants were planted under LD (14 h light from 5:00 to 17:00, 10 h darkness) and SD (9 h light from 8:00 to 17:00, 15 h darkness) conditions, respectively. Leaf samples from at least three sesame plants were collected every 4 h during a 24 h period (at 0:00, 4:00, 8:00, 12:00 16:00 and 20:00 every day) at the last week before flowering. The leaves were frozen in liquid nitrogen and total RNA was isolated immediately.

Totally, 132 sesame landraces from 13 counties in South Asia, Southeast Asia, East Asia and Central Asia were selected from sesame core-collections and planted in summer of Wuhan, China from 2015 to 2017 (Additional file 1: Table S3). The flowering date of each landrace was recorded. All the sesame samples were provided by the Oil Crops Research Institute, Chinese Academy of Agricultural Sciences, Wuhan, China.

### Overexpression of *SiCOL1* and *SiCOL2* in transgenic *Arabidopsis*

The binary vector pBI121 was digested by restriction enzymes *Sma* I and *Sac* I. We combined the amplified cDNA of *SiCOL1* and *SiCOL2* with the linear vector pBI121 using one step cloning kit (ClonExpress, Vazyme), and then transformed it into *Agrobacterium tumefaciens*. *Arabidopsis* was then transformed by the floral dip method [85]. Plasmid isolation was performed using the Plasmid DNA mini kit (Omega). The nucleotide sequencing was determined by Tsingke Company (Wuhan, China). The analysis of nucleotide sequence was done by the BioEdit [86] and DNAstar Lasergene (http://www.dnastar.com/t-dnastar-lasergene.aspx).

Kanamycin-resistant transgenic *Arabidopsis* T_0_ plants were regenerated, allowed to self-fertilize and T_1_ seeds were sown on medium containing kanamycin. Ten independently transformed kanamycin-resistant lines were self-fertilized and T_2_ seed collected from each individual. Then ten individuals of T_2_ generation were grown in LD condition (22 °C, 14 h photoperiod). Flowering time was measured as the number of days from sowing to the appearance of flower buds in the center of the plant rosette. In about 1 week before flowering, leaves of T_2_ lines were collected from the wild type *Arabidopsis* and individuals with overexpressed *SiCOL1* and *SiCOL2*.

### Expression analysis

Total mRNA was extracted using the RNA extraction kit EASYspin Plus Plant RNA Kit (Aidlab Biotechnologies, Beijing, China) according to the manufacturer’s instructions. The RNA was reverse-transcribed into cDNA using the iScript cDNA Synthesis kit (Bio-Rad, Hercules, USA). The quantitative real-time PCR (qRT-PCR) experiments were performed with gene-specific primers in the reaction system of SYBR Green Supermix (Bio-rad, USA) on the CFX384 Real-Time System (Bio-Rad) according to the manufacturer’s instructions. The qRT–PCR assay was performed in triplicate with independent individuals and the *actin* (*At3g18780*) and sesame *actin7* gene (*SIN_1006268*) were used as internal controls for *Arabidopsis* and sesame genes, respectively. The expression data of *SiCOL1*, *SiCOL2*, *SiFT* (*SIN_1009320*) and *FT* were quantified by the 2^-ΔΔCT^ method [87]. qRT-PCR primers used for sesame and transgenic *Arabidopsis* were listed in Additional file 1: Table S4. All primers were synthesized by Tsingke Company (Wuhan, China).

### Haplotype and network analyses

SNPs of *SiCOL1* in 132 landrace genomes were selected and downloaded from the SesameHapMap database (http://www.ncgr.ac.cn/SesameHapMap/) [13]. The *SiCOL1* sequence regions included coding region, promoter and intron. These 132 landraces were selected from 13 Asian countries, containing Afghanistan, Bangladesh, Burma, China, India, Japan, Nepal, Pakistan, Philippines, South Korea, Thailand, Uzbekistan, and Vietnam. Haplotypes of *SiCOL1* and *SiCOL2* in these landraces were generated by DNASP version 6.0 [88].

Ten accessions of the landraces were selected and their *SiCOL1* and *SiCOL2* genes were sequenced. The information of the accessions was available at Additional file 1: Table S3. There were two haplotypes of *SiCOL1* in these accessions -- Hap1 (S012, S016, S060, S062 and S075) and Hap15 (S050, S053, S054, S057 and S115 was Hap15). There were five haplotypes of *SiCOL2* in these accessions. The haplotype of *SiCOL2* in S050, S053, S057, S060 and S062 was Hap1. The haplotypes of *SiCOL2* in S012, S016, S054, S075 and S112 included Hap2, Hap3, Hap4 and Hap8. SNPs and Indels of these genes in the accessions were identified by aligning with ClustalX 2.0 [79]. Primers used in the PCR were provided in Additional file 1: Table S4.

The haplotype networks of *SiCOL1* and *SiCOL2* were constructed by mutational steps with NETWORK 4.6 [89]. The networks represented the genetic distance of DNA sequences or alleles and were mainly consist of circles of different sizes and colors as well as lines that linked the circles. The circle size was proportional to the number of samples within a given haplotype, and the lines between the haplotypes represented mutational steps between the alleles.

The distribution of *SiCOL1* haplotypes was showed in a map of Asia. The original map was downloaded and adapted from “https://commons.wikimedia.org/wiki/File:BlankMap-Asia.png“(Bytebear at the English language Wikipedia). This original map is licensed under the Creative Commons Attribution-Share Alike 3.0 Unported license, which allows us to share and adapt for free with proper attribution. *SiCOL1* haplotypes was indicated by different colors. The size of the circles was proportional to the number of sesame landraces.

## Additional file

Additional file 1: **Figure S1.** Phylogenetic relationships and structures of SiCOL proteins. **Figure S2.** Comparison of SiCOL1, SiCOL2 and CO protein sequences. **Figure S3.** Relative expression of *FT* in leaves of T_2_ transgenic *Arabidopsis* lines with overexpressed *SiCOL1* and *SiCOL2*. **Figure S4.** Relative expression of *SiCOL2* in different tissues and development stages of sesame. **Figure S5.** Relative diurnal expression of *SiCOL2* under LD and SD conditions. **Figure S6.** Nucleotide changes in the coding region of *SiCOL2* among cultivated sesame. **Figure S7.** Sequences of *SiCOL1* in ten sesame landraces. **Figure S8.** Haplotype network of *SiCOL2*. **Table S1.** Information of B-box gene family and CCT-containing gene family in sesame genome. **Table S2.** Days to flowering of *Arabidopsis* samples. **Table S3.** Information of the sesame landraces from Asia used in the present study. **Table S4.** Primers used in the qRT-PCR. (PDF 634 kb)

## Abbreviations

Aa: Amino acid
BLAST: Basic local alignment search tool
CCT: CONSTANS, CONSTANS-like, TIMING OF CAB EXPRESSION 1
CDD: Conserved domain database
COL: CONSTANS-like
FT: FLOWERING LOCUS T
GSDS: Gene structure display server
HMM: Hidden Markov model
LD: Long day
LGs: Linkage groups
MEME: Multiple Em for Motif Elicitation
NJ: Neighbor-joining
qRT-PCR: quantitative real-time polymerase chain reaction
QTLs: Quantitative trait loci
SD: Short day
SMART: Simple modular architecture research tool
SNP: Single nucleotide polymorphism
WGD: Whole genome duplication
