# Supplementary material for: Photoperiod response-related gene SiCOL1 contributes to flowering in sesame
Source: BMC Plant Biol. 2018 Dec 10;18:343. doi: 10.1186/s12870-018-1583-z (PMC6288898; doi:10.1186/s12870-018-1583-z)
Supplement: Supplementary file 1 — Figure S1. Phylogenetic relationships and structures of SiCOL proteins. Figure S2. Comparison of SiCOL1, SiCOL2 and CO protein sequences. Figure S3. Relative expression of FT in leaves of T2 transgenic Arabidopsis lines with overexpressed SiCOL1 and SiCOL2. Figure S4. Relative expression of SiCOL2 in different tissues and development stages of sesame. Figure S5. Relative diurnal expression of SiCOL2 under LD and SD conditions. Figure S6. Nucleotide changes in the coding region of SiCOL2 among cultivated sesame. Figure S7. Sequences of SiCOL1 in ten sesame landraces. Figure S8. Haplotype network of SiCOL2. Table S1. Information of B-box gene family and CCT-containing gene family in sesame genome. Table S2. Days to flowering of Arabidopsis samples. Table S3. Information of the sesame landraces from Asia used in the present study. Table S4. Primers used in the qRT-PCR. (PDF 634 kb) [file 12870_2018_1583_MOESM1_ESM.pdf]

Photoperiod response-related gene *SiCOL1* contributes to flowering in sesame

Rong Zhou[1], Pan Liu[1], Donghua Li[1], Xiurong Zhang[1*], Xin Wei[1,2*]

[1]Key Laboratory of Biology and Genetic Improvement of Oil Crops of the Ministry of Agriculture, Oil Crops Research Institute of the Chinese Academy of Agricultural Sciences, Wuhan, 430062, China

[2]College of Life Sciences, Shanghai Normal University, Shanghai, 200234, China

[*]Correspondence: xwei@shnu.edu.cn (XW) and zhangxr@oilcrops.cn (XZ)

Supplementary Figures

Figure S1 Phylogenetic relationships and structures of SiCOL proteins.

Figure S2 Comparison of SiCOL1, SiCOL2 and CO protein sequences.

Figure S3 Relative expression of *FT* in leaves of T_2 transgenic *Arabidopsis* lines with overexpressed *SiCOL1* and *SiCOL2*.

Figure S4 Relative expression of *SiCOL2* in different tissues and development stages of sesame.

Figure S5 Relative diurnal expression of *SiCOL2* under LD and SD conditions.

Figure S6 Nucleotide changes in the coding region of *SiCOL2* among cultivated sesame.

Figure S7 Sequences of *SiCOL1* in ten sesame landraces.

Figure S8 Haplotype network of *SiCOL2*.

Supplementary Tables

Table S1 Information of B-box gene family and CCT-containing gene family in sesame genome.

Table S2 Days to flowering of *Arabidopsis* samples.

Table S3 Information of the sesame landraces from Asia used in the present study.

Table S4 Primers used in the qRT-PCR.

Supplementary Figures

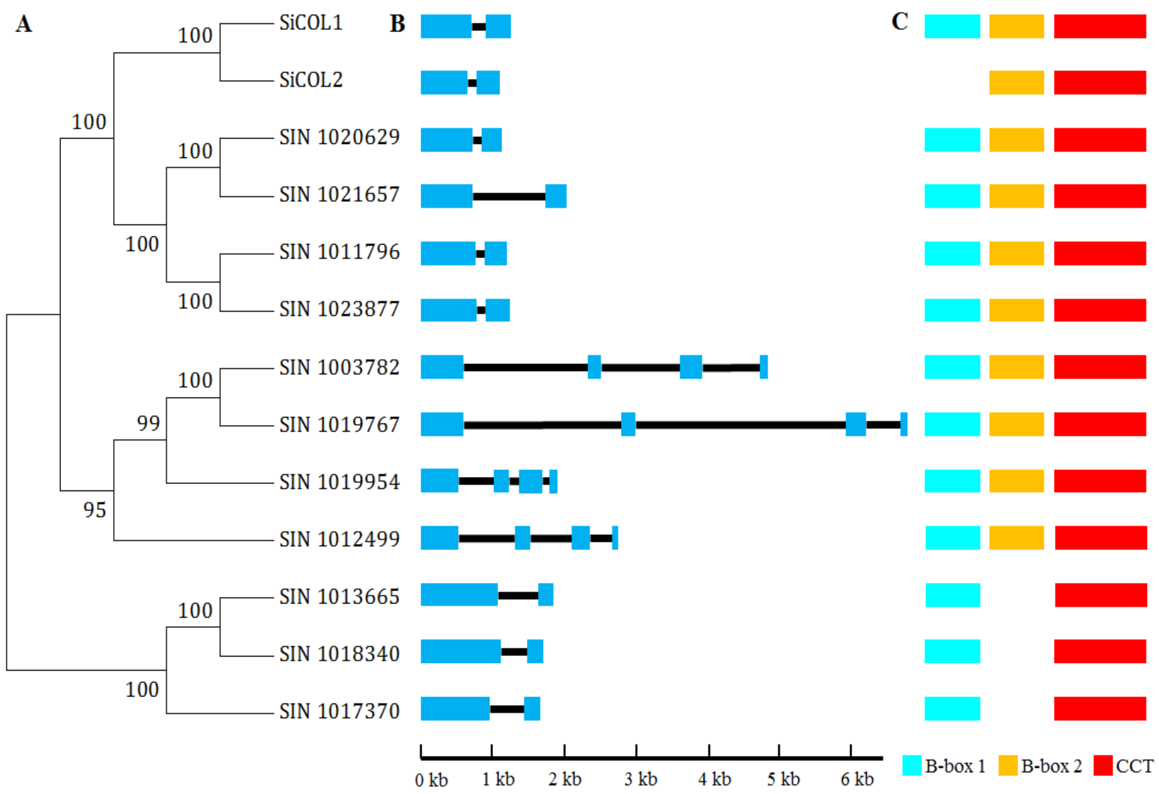

Figure S1 Phylogenetic relationships and structures of SiCOL proteins. A A NJ phylogenetic tree of the SiCOL proteins. Bootstrap values from 1,000 replicates are shown in the nodes. **B** The gene structure of *SiCOLs*. Blue boxes and black lines were exon and intron regions, respectively. **C** The domain structure of SiCOL proteins. Colorful boxes indicated B-box 1, B-box 2 and CCT domain, respectively.

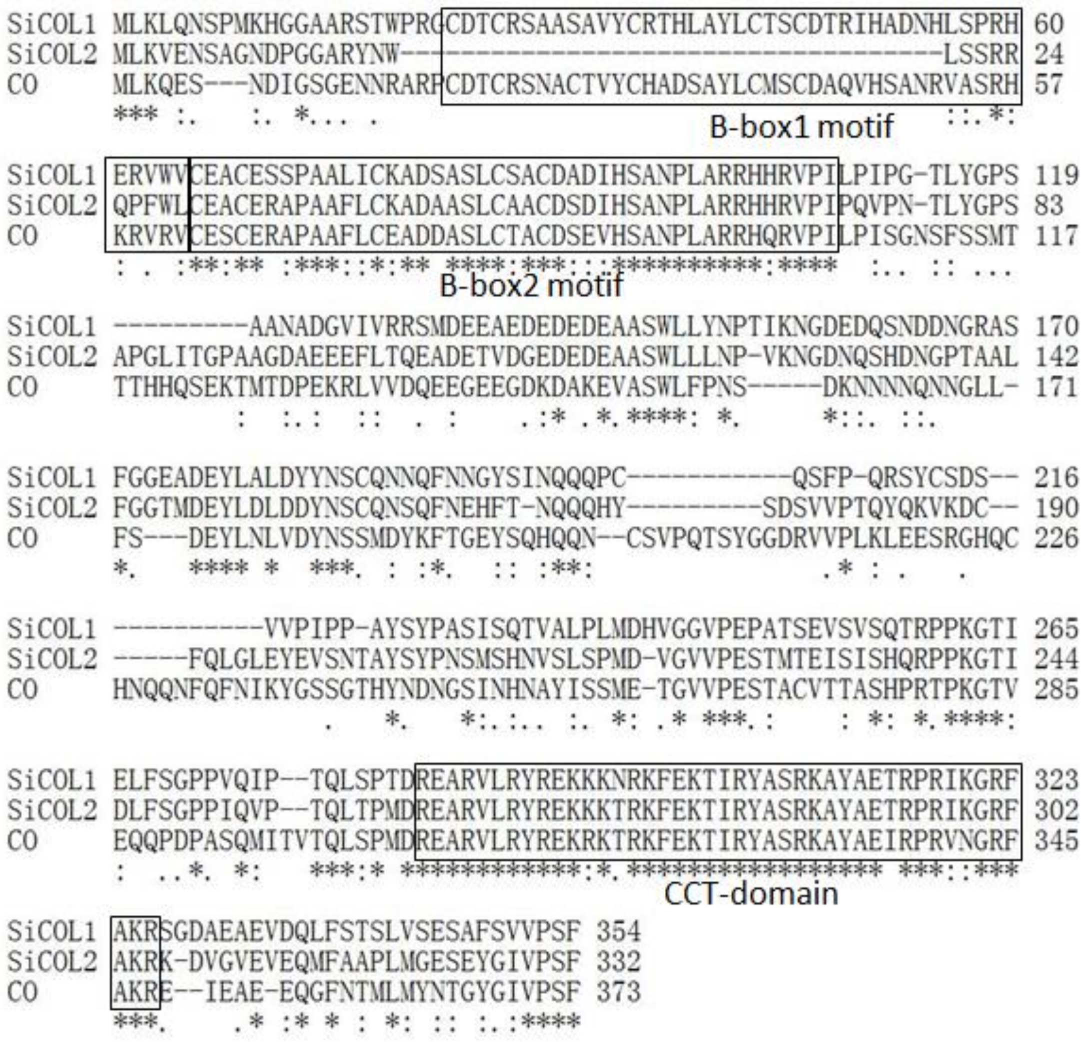

Figure S2 Comparison of SiCOL1, SiCOL2 and CO protein sequences. The B-box motifs and CCT domain were indicated. '*' indicates positions which have a single, fully conserved residue. ':' and '.' indicate strong and weak groups are fully conserved, respectively. These are all the positively scoring groups that occur in the Gonnet Pam250 matrix [79]. The strong and weak groups are defined as strong score > 0.5 and weak score ≤ 0.5, respectively.

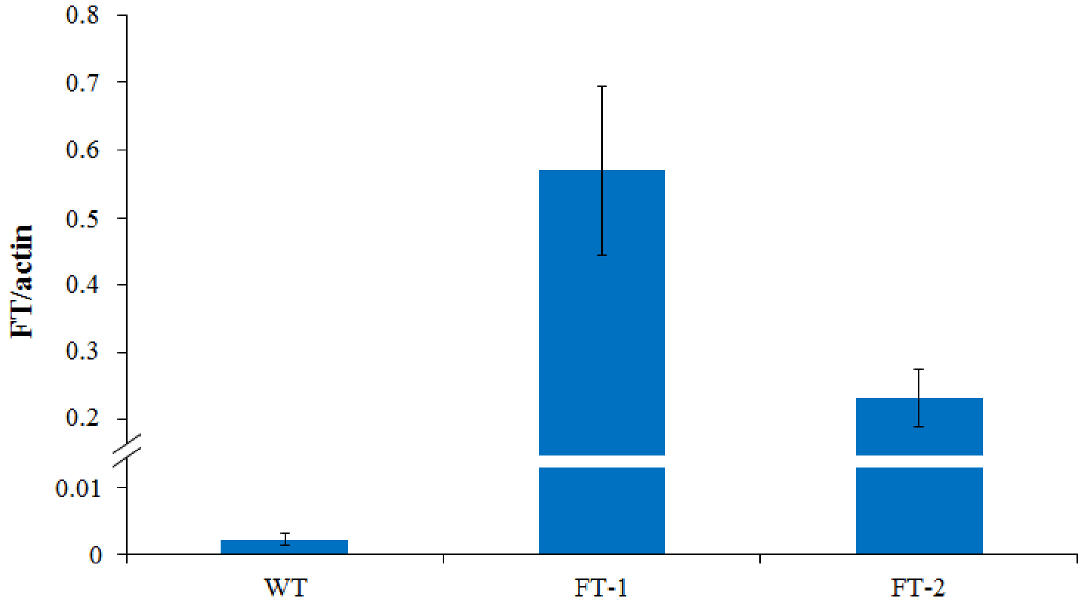

Figure S3 Relative expression of *FT* in leaves of T$_2$ transgenic *Arabidopsis* lines with overexpressed *SiCOL1* and *SiCOL2*. FT-1 and FT-2 indicate the *FT* in T$_2$ transgenic *Arabidopsis* lines with overexpressed *SiCOL1* and *SiCOL2*, respectively. Transcript abundance was quantified using qRT-PCR and expression levels were normalized using *actin* as a reference gene. The bar indicates standard deviation.

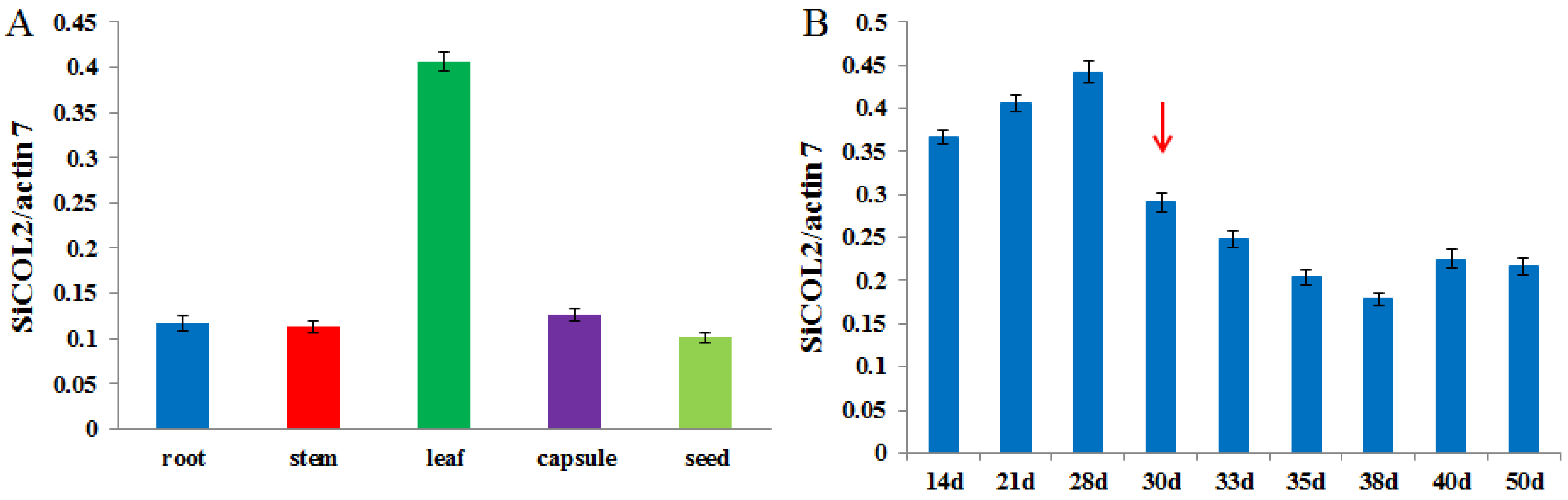

Figure S4 Relative expression of *SiCOL2* in different tissues and development stages of sesame. A Relative expression of *SiCOL2* in five tissues of sesame. **B** Relative expression of *SiCOL2* in leaves of different development stages. The red throw indicates that tiny flower buds begin to appear in the axil of sesame plants. Transcript abundance was quantified using qRT-PCR and expression levels were normalized using sesame *actin7* as a reference gene. The bar indicates standard deviation.

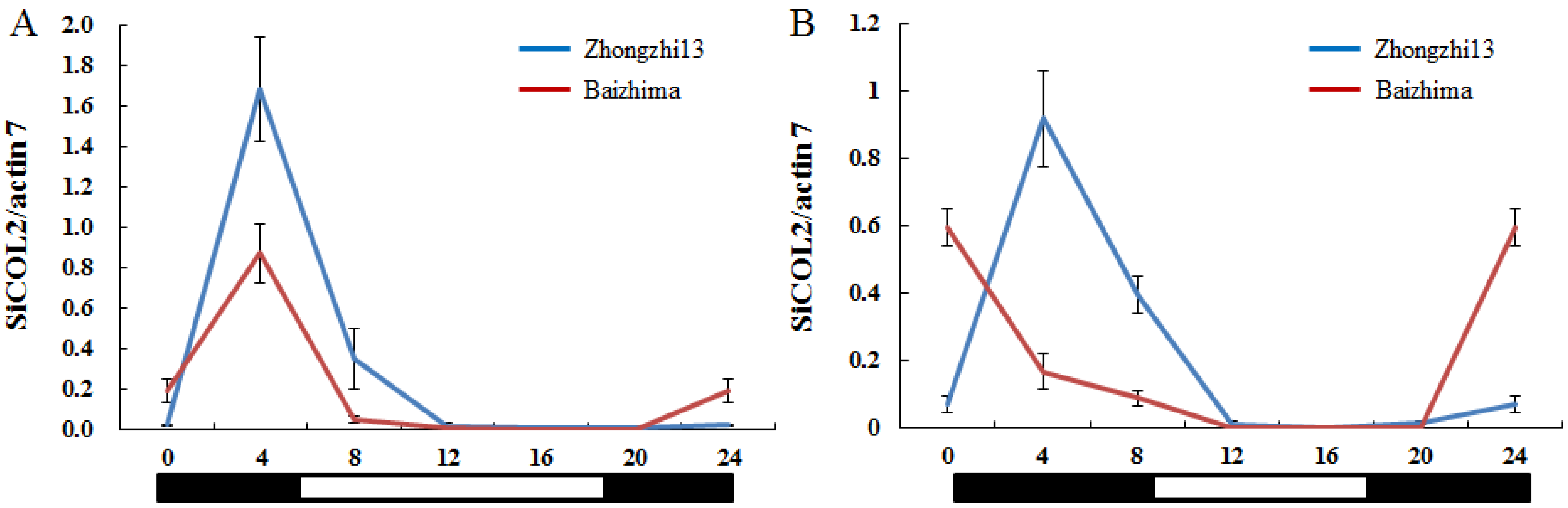

Figure S5 Relative diurnal expression of *SiCOL2* under LD and SD conditions. A Relative expression of *SiCOL2* under LD condition. **B** Relative expression of *SiCOL2* under SD condition. White boxes below the graphs indicate light periods and dark boxes indicate darkness. The expression data was normalized by sesame *actin7*. The bar indicates standard deviation.

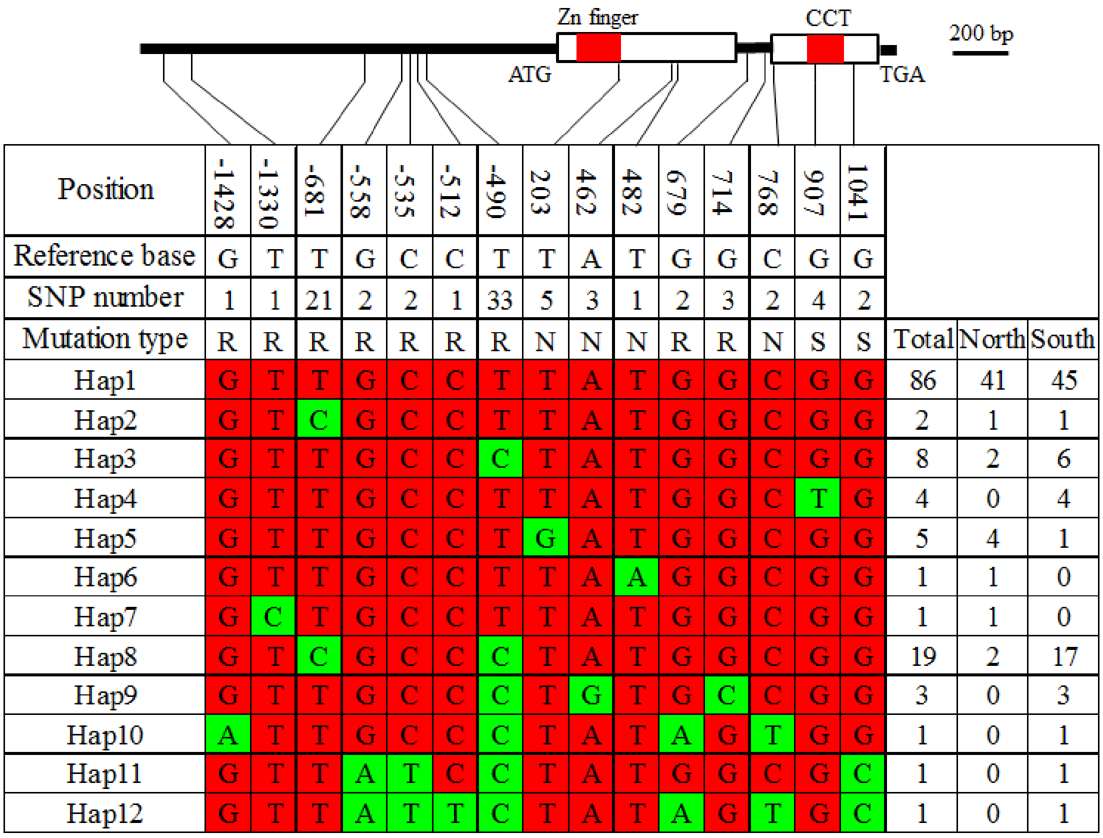

Position	-1428	-1330	-681	-558	-535	-512	-490	203	462	482	679	714	768	907	1041			
Reference base	G	T	T	G	C	C	T	T	A	T	G	G	C	G	G			
SNP number	1	1	21	2	2	1	33	5	3	1	2	3	2	4	2			
Mutation type	R	R	R	R	R	R	R	N	N	N	R	R	N	S	S	Total	North	South
Hap1	G	T	T	G	C	C	T	T	A	T	G	G	C	G	G	86	41	45
Hap2	G	T	C	G	C	C	T	T	A	T	G	G	C	G	G	2	1	1
Hap3	G	T	T	G	C	C	C	T	A	T	G	G	C	G	G	8	2	6
Hap4	G	T	T	G	C	C	T	T	A	T	G	G	C	T	G	4	0	4
Hap5	G	T	T	G	C	C	T	G	A	T	G	G	C	G	G	5	4	1
Hap6	G	T	T	G	C	C	T	T	A	A	G	G	C	G	G	1	1	0
Hap7	G	C	T	G	C	C	T	T	A	T	G	G	C	G	G	1	1	0
Hap8	G	T	C	G	C	C	C	T	A	T	G	G	C	G	G	19	2	17
Hap9	G	T	T	G	C	C	C	T	G	T	G	C	C	G	G	3	0	3
Hap10	A	T	T	G	C	C	C	T	A	T	A	G	T	G	G	1	0	1
Hap11	G	T	T	A	T	C	C	T	A	T	G	G	C	G	C	1	0	1
Hap12	G	T	T	A	T	T	C	T	A	T	A	G	T	G	C	1	0	1

Figure S6 Nucleotide changes in the coding region of *SiCOL2* among cultivated sesame. Reference base is the base in reference genome 'Zhongzhi13'. SNP number is the mutation number among the 132 landraces. R, S and N in mutation type indicate replacement, synonymous SNP and nonsynonymous SNP, respectively. Numbers in the right column are numbers of cultivars represented in every haplotypes. Total, South and North indicate total landraces, landraces from south group and landraces from north group, respectively. Variations that different from the reference bases are shown in green.

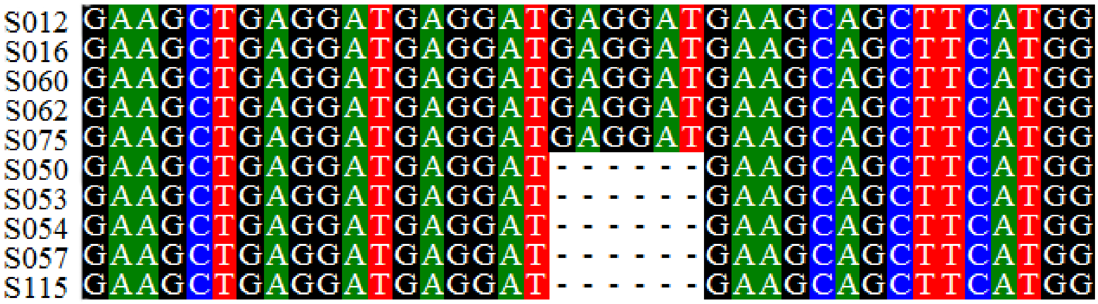

Figure S7 Sequences of *SiCOL1* in ten sesame landraces. The 6 bp deletion was found in the sequences of landraces with Hap15. The detailed information of the landraces was available at Table S3.

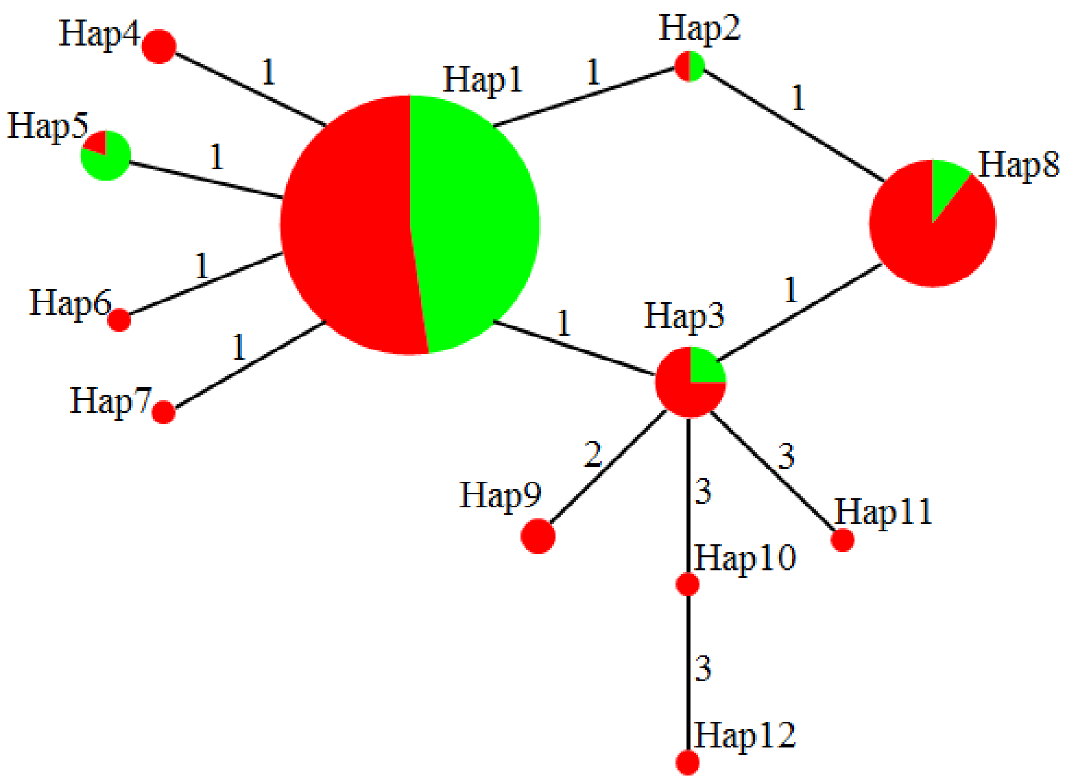

Figure S8 Haplotype network of *SiCOL2*. Haplotypes are showed by colored solid circles. Circle size is proportional to the quantity of samples within a given haplotype. Lines between haplotypes represent mutational steps between alleles. The numbers next to the lines indicate the nucleotide difference existed between the linked haplotypes. The red and blue color indicates landraces from south group and north group, respectively.

Supplementary Tables

Table S1 Information of B-box gene family and CCT-containing gene family in sesame genome.

ID	Gene	E-value	Score	Number	LG	Position
B-box1	SIN_1000235	4.70E-09	36.8	2	scaffold00498	508-8648
B-box2	SIN_1001371	2.00E-06	28.4	3	LG12	3725144-3725923
B-box3	SIN_1001451	0.0034	18.1	2	scaffold00165	24583-26061
B-box4	SIN_1002219	0.00064	20.4	2	LG3	3248013-3249699
B-box5	SIN_1003782	1.30E-08	35.5	2	LG16	4171550-4176377
B-box6	SIN_1004479	0.0019	18.9	2	LG11	12834357-12835660
B-box7	SIN_1004520	9.60E-14	51.8	2	LG10	16533928-16535789
B-box8	SIN_1004896	3.30E-07	30.9	1	LG15	2534501-2535610
B-box9	SIN_1009097	1.10E-12	48.4	2	LG3	23443287-23444467
B-box10	SIN_1011537	5.00E-13	49.5	2	LG7	3254481-3255641
B-box11	SIN_1011796	3.60E-10	40.4	2	LG5	17536289-17537482
B-box12	SIN_1012499	1.20E-09	38.8	3	LG6	1093823-1096564
B-box13	SIN_1013522	0.00064	20.4	1	LG5	5718885-5729717
B-box14	SIN_1013665	6.20E-09	36.5	1	LG1	10021319-10023171
B-box15	SIN_1014520	6.60E-07	30	3	LG12	247769-248701
B-box16	SIN_1014730	5.00E-13	49.5	2	LG8	20586653-20588389
B-box17	SIN_1014743	2.10E-16	60.3	3	LG8	20489435-20490511
B-box18	SIN_1015960	6.30E-12	46	3	LG3	18348540-18351621
B-box19	SIN_1016011	0.0052	17.5	2	LG5	8155957-8157602
B-box20	SIN_1016901	3.20E-05	24.6	3	LG16	1466366-1466815
B-box21	SIN_1017370	1.00E-07	32.5	1	LG3	11310021-11311664
B-box22	SIN_1017513	2.30E-06	28.2	2	LG3	9802259-9802930

B-box23	SIN_1018340	1.70E-08	35	1	LG2	16108396-16110105
B-box24	SIN_1019210	5.80E-13	49.3	2	LG10	5985140-5987670
B-box25	SIN_1019417	6.10E-13	49.3	2	LG14	3894339-3897686
B-box26	SIN_1019767	1.30E-07	32.2	2	LG8	1838327-1845107
B-box27	SIN_1019889	3.30E-09	37.3	2	LG8	2936072-2937312
B-box28	SIN_1019954	0.0028	18.4	2	LG5	13943158-13945055
B-box29	SIN_1019960	4.70E-06	27.2	1	LG5	14074758-14075066
B-box30	SIN_1019994	0.0083	16.8	3	LG5	14864233-14865050
B-box31	SIN_1020140	0.0016	19.1	1	LG5	16847867-16854721
B-box32	SIN_1020629	3.00E-13	50.2	2	LG6	18213252-18214375
B-box33	SIN_1020739	0.0006	20.5	1	LG6	17458017-17461708
B-box34	SIN_1021657	1.50E-13	51.2	2	LG1	930153-932165
B-box35	SIN_1022519	5.60E-10	39.8	2	LG6	22132686-22135112
B-box36	SIN_1023739	0.0024	18.5	1	LG2	11013816-11019898
B-box37	SIN_1023877	8.70E-13	48.8	2	LG2	9655751-9656982
CCT1	SIN_1002800	5.10E-18	65.4	1	LG10	2646228-2649123
CCT2	SIN_1003172	4.70E-17	62.3	1	LG14	18264-20779
CCT3	SIN_1003782	4.60E-16	59.2	1	LG16	4171550-4176377
CCT4	SIN_1004027	1.50E-18	67.2	1	LG13	3405604-3408756
CCT5	SIN_1004233	1.70E-10	41.4	2	LG15	1426384-1427703
CCT6	SIN_1004896	1.80E-17	63.7	1	LG15	2534501-2535610
CCT7	SIN_1005900	4.30E-17	62.5	1	LG4	8619497-8624397
CCT8	SIN_1005989	7.60E-14	52.1	1	LG8	15096112-15104658
CCT9	SIN_1006875	5.90E-17	62.1	1	LG12	2589677-2592703
CCT10	SIN_1007726	2.60E-05	24.8	1	LG5	2074223-2076532
CCT11	SIN_1009194	0.00078	20	2	LG7	9062162-9064884

CCT12	SIN_1009237	2.40E-17	63.3	1	LG7	9348457-9352056
CCT13	SIN_1011285	2.30E-16	60.2	1	LG11	13633366-13639025
CCT14	SIN_1011796	2.00E-18	66.7	1	LG5	17536289-17537482
CCT15	SIN_1012499	1.70E-16	60.6	1	LG6	1093823-1096564
CCT16	SIN_1013665	1.30E-17	64.2	1	LG1	10021319-10023171
CCT17	SIN_1014239	3.40E-17	62.8	1	LG12	2075343-2076486
CCT18	SIN_1014633	1.80E-13	50.9	1	LG8	21100088-21100747
CCT19	SIN_1015297	7.30E-16	58.5	1	LG3	16730565-16737598
CCT20	SIN_1015513	1.90E-18	66.8	1	LG6	8148404-8150002
CCT21	SIN_1016429	1.50E-17	64	2	LG4	9154308-9156996
CCT22	SIN_1017370	4.70E-18	65.5	1	LG3	11310021-11311664
CCT23	SIN_1017486	1.80E-15	57.3	1	LG3	9994680-10000625
CCT24	SIN_1017911	4.30E-19	68.9	1	LG1	7804119-7805369
CCT25	SIN_1018340	5.60E-18	65.3	1	LG2	16108396-16110105
CCT26	SIN_1019767	2.00E-16	60.4	1	LG8	1838327-1845107
CCT27	SIN_1019889	9.00E-18	64.7	1	LG8	2936072-2937312
CCT28	SIN_1019954	1.30E-16	61	1	LG5	13943158-13945055
CCT29	SIN_1020629	5.80E-18	65.3	1	LG6	18213252-18214375
CCT30	SIN_1021657	5.50E-18	65.3	1	LG1	930153-932165
CCT31	SIN_1023877	4.60E-18	65.6	1	LG2	9655751-9656982
CCT32	SIN_1024178	6.40E-12	45.9	1	LG9	4998282-4999672
CCT33	SIN_1024493	1.70E-18	67	1	LG9	2745883-2747059
CCT34	SIN_1026120	8.90E-16	58.3	1	LG10	15054383-15058219
CCT35	SIN_1026121	1.50E-15	57.5	1	LG10	15061838-15067703
CCT36	SIN_1026433	8.00E-17	61.6	1	LG8	13223513-13226854

Table S2 Days to flowering of *Arabidopsis* samples.

Arabidopsis samples	Days to flowering (d)									
	line 1	line 2	line 3	line 4	line 5	line 6	line 7	line 8	line 9	line 10
35S::SiCOL1	29	27	25	28	30	26	25	25	29	30
	27	26	30	30	28	29	28	27	28	29
	30	26	27	28	30	28	27	28	28	31
	31	27	29	26	32	29	28	26	31	28
	28	27	28	28	28	28	26	25	28	27
	26	28	26	27	27	25	28	28	30	28
	28	29	28	28	32	29	29	26	28	29
	26	30	27	28	31	26	28	29	28	31
	32	27	26	27	29	28	25	27	29	29
	29	26	27	28	29	24	29	28	26	28
35S::SiCOL2	28	31	27	29	29	30	32	29	32	34
	27	32	29	31	31	31	29	28	30	30
	27	29	27	28	32	30	29	31	30	31
	30	30	29	30	31	31	30	25	28	31
	28	31	28	27	33	28	31	27	30	32
	31	30	30	30	31	29	30	30	29	30
	28	33	28	31	30	31	31	29	30	33
	32	30	28	30	29	32	32	26	31	29
	30	32	30	31	30	30	29	29	30	32
	27	30	29	30	32	31	32	27	31	30
empty vector	31	34	33	30	36	38	32	32	35	37
	34	33	35	31	37	35	33	33	37	35
	33	35	31	30	33	36	34	31	36	38

	32	36	32	31	36	34	31	33	33	33
	33	32	33	33	36	37	33	35	37	39
	30	34	31	31	35	33	31	33	33	34
	33	33	33	34	33	34	35	34	35	32
	31	34	32	30	34	33	32	33	32	34
	33	35	33	32	33	32	33	31	31	36
	32	31	32	31	32	34	31	33	33	33
Col-0	31	32	34	32	34	35	31	33	33	35

Ten individuals of ten T_2 lines of *35S::SiCOL1*, *35S::SiCOL2* and empty vector as well as ten Col-0 individuals were grown in LD condition and the flowering time of each individual was recorded. The days to flowering was measured as the number of days from sowing to the appearance of flower buds in the center of the plant rosette.

Table S3 Information of the sesame landraces from Asia used in the present study.

ID	Name	Origin	Longitude(º)	Latitude(º)	Group	Flowering date （d）			SiCOL1 hap	SiCOL2 hap
						2015	2016	2017		
S001	726	Afghanistan	69.10E	34.31N	North	48	55	56	6	8
S002	725	Afghanistan	69.10E	34.31N	North	46	56	46	6	8
S003	835-1	Bangladesh	90.24E	23.42N	South	52	51	56	1	1
S004	Ses-11	Bangladesh	90.24E	23.42N	South	52	55	55	1	1
S005	Ses-12	Bangladesh	90.24E	23.42N	South	40	46	43	4	8
S006	Heizhima	Bangladesh	90.24E	23.42N	South	47	43	52	10	8
S007	ZM11-17	Burma	96.09E	16.48N	South	47	51	50	1	1
S008	ZM11-18	Burma	96.09E	16.48N	South	52	51	51	1	1
S009	Tha Dun Byu	Burma	96.06E	19.45N	South	48	54	52	14	1
S010	Potepye	Burma	96.06E	19.45N	South	42	54	44	2	1
S011	Patiae-1	Burma	96.50E	21.58N	South	44	55	46	1	1
S012*	Magwe 719	Burma	96.50E	21.58N	South	48	54	47	1	4
S013	Maeshae	Burma	95.39E	19.08N	South	48	52	47	3	1
S014	Sinyadanar	Burma	95.39E	19.08N	South	58	61	56	1	4
S015	Potepye	Burma	95.39E	19.08N	South	46	53	47	15	8
S016*	Heizhima	Burma	96.09E	16.48N	South	59	54	57	1	3
S017	Baicao	China	114.90E	33.44N	North	39	48	41	15	1
S018	Tanma	China	113.69E	31.26N	South	42	48	47	15	1
S019	Zhima	China	89.18E	42.94N	North	36	40	37	14	1
S020	Zhima	China	89.18E	42.94N	North	39	38	40	15	1
S021	Hezhima	China	109.89E	31.08N	South	40	44	42	14	1
S022	Heizhima	China	105.38E	31.23N	South	51	55	48	1	1
S023	Zhima	China	116.35E	23.54N	South	52	53	48	6	1

S024	Zhima	China	121.65E	42.01N	North	36	42	41	15	1
S025	Jiaopanzhima	China	111.78E	37.14N	North	37	43	41	15	1
S026	Laozhima	China	110.68E	31.75N	South	47	52	48	15	1
S027	Qingchunheizhima	China	121.37E	31.11N	South	42	46	48	6	1
S028	Liujiaobaizhima	China	121.37E	31.11N	South	53	53	48	1	1
S029	Huizhima	China	106.10E	25.17N	South	44	50	49	1	1
S030	Huangzhima	China	106.85E	22.35N	South	46	53	51	6	1
S031	Heizhima	China	109.27E	22.69N	South	46	56	50	6	1
S032	Baizhima	China	109.84E	19.05N	South	53	64	57	1	1
S033	Zhima	China	113.81E	36.07N	North	48	53	47	15	1
S034	Sitongzhima	China	113.35E	34.51N	North	43	55	50	15	1
S035	Baizhima	China	113.96E	35.05N	North	40	42	42	14	1
S036	Yingshangzhima	China	116.25E	32.64N	North	46	50	44	14	1
S037	Zhima	China	109.29E	37.96N	North	50	55	54	15	1
S038	Hongzhima	China	106.16E	33.33N	North	39	52	44	15	1
S039	Heizhima	China	115.21E	29.83N	South	49	54	47	1	2
S040	Beisanxie	China	111.32E	30.77N	South	59	61	55	1	1
S041	Zhima	China	116.55E	29.90N	South	45	54	45	1	1
S042	Zhima	China	120.83E	37.30N	North	40	50	46	15	1
S043	Bacha	China	123.49E	43.51N	North	39	35	32	15	1
S044	Huangzhima	China	108.12E	28.26N	South	50	55	54	1	1
S045	Bawangbian	China	117.06E	39.38N	North	38	41	43	15	1
S046	Zhima	China	115.02E	37.21N	North	41	46	44	15	1
S047	Laohongzhima	China	111.48E	33.14N	North	37	53	42	15	1
S048	Yeersan	China	114.63E	32.96N	North	38	46	44	15	1
S049	Baizhima	China	125.08E	45.51N	North	27	33	32	14	1

S050*	Bacha	China	122.77E	45.34N	North	32	35	36	15	1
S051	Deleilei	China	119.42E	32.39N	North	41	40	42	15	1
S052	Zhima	China	95.26E	25.22N	South	44	53	48	15	1
S053*	Xinyangbaizhima	China	120.26E	33.77N	North	47	50	46	15	1
S054*	Baizhima	China	120.14E	33.39N	North	41	46	44	15	2
S055	Baizhima	China	119.57E	31.75N	South	44	46	44	15	1
S056	Heizhima	China	120.46E	33.20N	North	44	51	51	6	5
S057*	Chama	China	121.08E	32.09N	North	41	46	43	15	1
S058	Baizhima	China	116.09E	25.09N	South	42	49	46	1	1
S059	Heizhima	China	118.14E	24.74N	South	41	44	42	1	1
S060*	Heizhima	China	117.60E	26.22N	South	41	42	43	1	1
S061	Zhima	China	100.56E	25.48N	South	48	55	54	1	1
S062*	Tainanheixuan2hao	China	120.31E	23.31N	South	52	65	57	1	1
S063	Zhima	China	122.07E	46.06N	North	35	40	43	15	1
S064	Baizhima	China	109.84E	19.05N	South	65	56	71	1	3
S065	C-50	India	72.12E	26.20N	South	65	71	55	13	1
S066	Ty-13	India	71.09E	23.14N	South	62	67	57	1	1
S067	Phule til 1	India	73.42E	22.43N	South	54	68	55	1	1
S068	Gouri	India	76.02E	23.17N	South	48	57	48	15	1
S069	Methila	India	79.19E	28.10N	South	48	53	46	1	1
S070	Hnan-ni	India	77.22E	24.34N	South	44	54	46	9	1
S071	Sinya Dana-2	India	75.58E	21.06N	South	52	59	55	1	1
S072	Sinya Dana-3	India	75.58E	21.06N	South	46	55	54	8	8
S073	EC—343404	India	77.32E	10.27N	South	42	53	47	6	8
S074	EC—342783	India	81.34E	17.20N	South	53	61	55	1	8
S075*	EC—355640	India	87.31E	22.55N	South	40	41	43	1	8

S076	EC－355666	India	84.02E	20.46N	South	40	45	46	1	8
S077	EC－357315	India	89.28E	26.29N	South	55	61	60	1	8
S078	TC-25	India	71.34E	26.11N	South	54	62	55	1	8
S079	865	India	69.50E	23.42N	South	53	76	56	5	12
S080	874	India	75.02E	27.06N	South	58	53	63	1	8
S081	891	India	72.38E	26.33N	South	45	62	46	11	8
S082	847	India	72.29E	25.56N	South	48	53	47	12	3
S083	10(27)	Japan	136.50E	35.12N	North	47	53	48	14	1
S084	110(1)	Japan	136.50E	35.12N	North	48	56	52	15	6
S085	TKV-334(22)	Japan	136.50E	35.12N	North	40	46	45	14	1
S086	723(38)	Japan	136.50E	35.12N	North	43	49	47	14	5
S087	323(4)	Japan	136.50E	35.12N	North	39	41	44	15	1
S088	384(7)	Japan	136.50E	35.12N	North	50	47	46	15	1
S089	342(5)	Japan	136.50E	35.12N	North	48	55	47	15	1
S090	TKV-726(16)	Japan	136.50E	35.12N	North	52	55	47	15	5
S091	301(3)	Japan	136.50E	35.12N	North	43	37	44	15	1
S092	Sursari Kall Til-20	Nepal	85.19E	27.42N	South	58	59	59	6	1
S093	697	Nepal	85.19E	27.42N	South	70	91	76	1	8
S094	1018	Pakistan	73.04E	31.25N	South	48	54	46	15	8
S095	1139	Pakistan	72.56E	33.30N	South	53	71	56	16	3
S096	1131	Pakistan	73.04E	31.25N	North	51	75	47	7	3
S097	His-173	Philippines	120.35E	17.20N	South	48	55	55	1	1
S098	His-167	Philippines	120.35E	17.20N	South	50	53	46	15	1
S099	Isi-12	Philippines	120.17E	15.54N	South	73	87	68	12	1
S100	His-167	Philippines	120.35E	17.20N	South	45	48	46	6	1
S101	Shuiyuan117	South Korea	127.14E	37.04N	North	41	45	46	15	3

S102	Nian	South Korea	125.48E	35.38N	North	46	46	46	15	5
S103	Zhenzhu	South Korea	126.58E	37.33N	North	39	38	38	15	1
S104	Danbai	South Korea	126.58E	37.33N	North	41	41	42	15	1
S105	Andong	South Korea	127.48E	35.34N	North	37	41	42	15	1
S106	Guangchan	South Korea	126.58E	37.33N	North	40	44	41	1	1
S107	Anchan	South Korea	126.58E	37.33N	North	37	40	46	15	1
S108	Shuiyuan117	South Korea	127.14E	37.04N	North	42	44	45	15	1
S109	Rucheng	South Korea	127.34E	36.05N	North	47	44	46	15	1
S110	Jinback	South Korea	127.07E	34.58N	North	41	44	47	15	1
S111	Yeoju-1	South Korea	127.32E	37.14N	North	40	43	44	15	7
S112	84013	Thailand	99.10E	18.34N	South	53	60	49	1	1
S113	MK60	Thailand	100.33E	18.19N	South	47	61	47	1	5
S114	Boder Racet	Thailand	100.31E	13.45N	South	84	92	75	1	1
S115*	393-3Bo	Thailand	101.01E	15.53N	South	46	52	47	15	4
S116	MKS-II-82266	Thailand	100.31E	13.45N	South	72	82	63	1	4
S117	Boder Racet	Thailand	100.31E	13.45N	South	46	56	47	1	8
S118	VIR 80	Uzbekistan	69.13E	41.16N	North	41	41	38	15	1
S119	VIR 825	Uzbekistan	69.13E	41.16N	North	42	52	41	15	1
S120	VIR 896	Uzbekistan	69.13E	41.16N	North	41	34	39	15	1
S121	Tashigan122	Uzbekistan	69.13E	41.16N	North	44	50	51	15	1
S122	Heizhima	Vietnam	103.53E	21.57N	South	44	51	47	14	1
S123	Heizhima	Vietnam	105.58E	21.48N	South	41	44	47	1	1
S124	Zhima21	Vietnam	105.51E	21.02N	South	50	45	46	1	8
S125	Zhima20	Vietnam	106.03E	17.52N	South	44	46	46	15	10
S126	Zhima12	Vietnam	107.51E	11.26N	South	65	85	66	1	9
S127	Zhima35	Vietnam	108.31E	12.19N	South	66	81	65	1	9

S128	Zhima52	Vietnam	107.37E	11.08N	South	67	86	69	1	8
S129	Zhima105	Vietnam	107.54E	13.56N	South	53	61	53	1	3
S130	Zhima30	Vietnam	105.01E	21.14N	South	46	52	48	1	9
S131	Zhima40	Vietnam	106.51E	21.27N	South	42	40	41	15	3
S132	Zhima	Vietnam	106.11E	17.47N	South	44	55	48	1	11

*: Accessions that *SiCOL1* and *SiCOL2* were sequenced.

Table S4 Primers used in the qRT-PCR.

Type	Species	Gene	Primer name	Sequence
qRT-PCR	sesame	*SiCOL1*	SiCOL1-F	AACGCTGACGGAGTC
			SiCOL1-R	CTTGATTGTAGGGTTGTAT
		SiCOL2	SiCOL2-F	GCTCTTTGGTGGAACG
			SiCOL2-R	CGCTGTCGCTGTAATG
		SiFT	SiFT-F	AGAAGTGAGCAATGGGTG
			SiFT-R	AAGAAGGTGCGGAGGT
		Siactin7	Siactin7-F	CTGTCAACAGAATTGGGTG
			Siactin7-R	GCAACTGGGATGATATGG
	Arabidopsis	*CO*	CO-F	ATTTCGTGCTGGTTGA
			CO-R	GGGACTAAGAAGGCTAT
		FT	FT-F	CCCTGCTACAACTGGAA
			FT-R	CAAGCTGTCGAAACAATAT
		actin	actin-F	CCCGCTATGTATGTCGCCA
			actin-R	AACCCTCGTAGATTGGCACAG
PCR	sesame	*SiCOL1*	SiCOL1-F	TTGCCACCATTACCTT
			SiCOL1-R	CAAATCCCGACCTCTT
		SiCOL2	SiCOL2-F	TCTTGCCATCTGTCGT
			SiCOL2-R	CTTGGTCCAACCATTT
